# Microstructures and Isothermal Oxidation of the Alumina Scale Forming Nb_1.45_Si_2.7_Ti_2.25_Al_3.25_Hf_0.35_ and Nb_1.35_Si_2.3_Ti_2.3_Al_3.7_Hf_0.35_ Alloys

**DOI:** 10.3390/ma12050759

**Published:** 2019-03-05

**Authors:** Mohammad Ghadyani, Claire Utton, Panos Tsakiropoulos

**Affiliations:** Department of Materials Science and Engineering, Sir Robert Hadfield Building, University of Sheffield, Mappin Street, Sheffield S1 3JD, UK; m.ghadyani@sheffield.ac.uk (M.G.); c.utton@sheffield.ac.uk (C.U.)

**Keywords:** Nb-silicide based alloys, high entropy alloys, coatings, intermetallics, pest oxidation, high temperature oxidation, complex concentrated alloys, multi-principle element alloys

## Abstract

Coating system(s) will be required for Nb-silicide based alloys. Alumina forming alloys that are chemically compatible with the Nb-silicide based alloy substrate could be components of such systems. The intermetallic alloys Nb_1.45_Si_2.7_Ti_2.25_Al_3.25_Hf_0.35_ (MG5) and Nb_1.35_Si_2.3_Ti_2.3_Al_3.7_Hf_0.35_ (MG6) were studied in the cast, heat treated and isothermally oxidised conditions at 800 and 1200 °C to find out if they are αAl_2_O_3_ scale formers. A (Al/Si)_alloy_ versus Nb/(Ti + Hf)_alloy_ map, which can be considered to be a map for Multi-Principle Element or Complex Concentrated Nb-Ti-Si-Al-Hf alloys, and a [Nb/(Ti + Hf)]_Nb5Si3_ versus [Nb/(Ti + Hf)]_alloy_ map were constructed making use of the alloy design methodology NICE and data from a previously studied alloy, and were used to select the alloys MG5 and MG6 that were expected (i) not to pest, (ii) to form αAl_2_O_3_ scale at 1200 °C, (iii) to have no solid solution, (iv) to form only hexagonal Nb_5_Si_3_ and (v) to have microstructures consisting of hexagonal Nb_5_Si_3_, Ti_5_Si_3_, Ti_5_Si_4_, TiSi silicides, and tri-aluminides and Al rich TiAl. Both alloys met the requirements (i) to (v). The alumina scale was able to self-heal at 1200 °C. Liquation in the alloy MG6 at 1200 °C was linked with the formation of a eutectic like structure and the TiAl aluminide in the cast alloy. Key to the oxidation of the alloys was the formation (i) of “composite” silicide grains in which the γNb_5_Si_3_ core was surrounded by the Ti_5_Si_4_ and TiSi silicides, and (ii) of tri-aluminides with high Al/Si ratio, particularly at 1200 °C and very low Nb/Ti ratio forming in-between the “composite” silicide grains. Both alloys met the “standard definition” of high entropy alloys (HEAs). Compared with HEAs with bcc solid solution and intermetallics, the VEC values of both the alloys were outside the range of reported values. The parameters VEC, Δχ and δ of Nb-Ti-Si-Al-Hf coating alloys and non-pesting Nb-silicide based alloys were compared and trends were established. Selection of coating alloys with possible “layered” structures was discussed and alloy compositions were proposed.

## 1. Introduction

Nb-silicide based alloys (or Nb-silicide in situ composites) are new materials and strong candidates for structural applications in gas turbines at high temperatures where the surface temperature of the alloy should not exceed 1400 °C. Like the Ni-based superalloys that are used in the latest generation of gas turbines, the Nb-silicide based alloys will require a coating [1,2] but must have adequate oxidation resistance to “survive” in case of failure of the coating. Unlike the Ni-based superalloys that are not prone to pest oxidation, the Nb-silicide based alloys will also need to have good resistance to pest oxidation. Contamination of Nb-silicide based alloys by interstitial elements must be controlled.

For high temperature applications, alloy developers aspire to discover alloys that can form compact protective self-healing oxide layer in which the diffusion of metal cations is inhibited (very slow). For Nb-silicide based alloys, coating system with alumina scale forming bond coat compatible with the substrate alloy is desirable [3]. An aim of this paper is to provide an insight into the design and selection of metallic materials for a coating system for Nb-silicide based alloys.

Some Nb-based alloys (not Nb-silicide based alloys) with high concentrations of Ti, Al, and Cr have good oxidation resistance in the 800–1200 °C range, approaching that of the oxidation-resistant superalloys [4]. In Nb-silicide based alloys the alloying additions of (i) Al, B, Cr, Fe, Ge, Hf, Si, Sn or Ti individually and in synergy have a strong effect on the compositions and properties of phases, for example see reference [5,6,7,8,9], and can improve the oxidation resistance of the alloys significantly [10,11,12,13,14,15,16,17], (ii) B, Ge or Sn are very effective for suppressing pest oxidation in Nb-silicide based alloys [15,16] and (iii) refractory metals significantly improve high temperature strength and creep [1], are not detrimental to oxidation at low concentrations [11,18], and have a strong effect on the composition of the Nb_ss_ [9,19].

A successful coating for Nb-silicide based alloy must demonstrate (i) low growth rate of the protective oxide layer, (ii) resistance to cracking, (iii) low evaporation rates of the protective oxide and other coating constituents, (iv) minimum coating-substrate interdiffusion and (v) self-healing ability. The self-healing ability of a coating is related to the preservation of an active reservoir of protective-oxide-forming substance in the coating. In the last two decades, researchers have studied silicide or aluminide based coatings that were applied on “selected” substrate alloys using mainly pack cementation, for example see references [20,21,22]. Currently, no coating for Nb-silicide based alloys that satisfies (i) to (v) is available. 

Coatings are available to protect refractory metal (RM) alloys (not RM-silicide based alloys) against oxidation [4]. They include (a) intermetallic compounds that form compact or glassy oxide layers, (b) alloys that form compact oxide layers, (c) noble metals (e.g., Pt, Ir) that resist oxidation, and (d) stable oxides that provide a physical barrier to the penetration of oxygen. 

Silicide(s) and/or aluminide(s) are desirable intermetallic compounds in coating alloys. Of particular interest are Al rich aluminides which can form a layer of crystalline Al_2_O_3_ because of the high activity of Al relative to the more noble constituent(s). At high temperatures D_Al_ >> D_O_ (D_i_ is diffusivity of element i = Al, O) and hence the outward migration of Al is expected to determine the oxidation rate constant [23]. When simple silicide or aluminide coatings were applied on refractory metal alloys, coating-substrate interdiffusion was the important degradation process of the coating in air or near 1370 °C and 1 atm pressure [24]. Even in the absence of cracking, a protective coating would ultimately fail due to the inevitable progress of interdiffusion with the substrate particularly at high temperatures. Different mechanism(s) of deterioration operate depending on the substrate/coating system and the process used for the application of coating (see below). 

Oxidation-resistant, non-structural alloys of the Nb-Ti-Al-Cr system, have been used to coat refractory metal alloys with some success. Such coatings could deform without cracking, and offered protection against foreign-object damage [24]. Also, Nb-Ti-Al based alloys with high concentrations of Al were reported to have excellent oxidation resistance [25]. 

An understanding of the microstructures that govern the performance of materials used in coatings is needed to provide a sound basis for developing advanced coating systems for a particular family of substrates. The performance of most coatings depends on defects introduced during the application of the coating, on random defects (cracks and fissures) and interdiffusion. Diffusion barriers can be used to minimise coating–substrate reaction. Hairline cracks and fissures in silicide coatings applied on Nb tend to govern life at atmospheric pressure, while at low pressure such defects tend to have little effect [26]. 

The type, number and distribution of defects and interdiffusion profiles are interrelated respectively with the coating process and substrate used. In the absence of defects, coating life is limited by diffusional processes at low and high temperature and by evaporation or melting at ultra-high temperatures. At temperatures above approximately 1650 °C, the evaporation rate of SiO_2_ becomes significant, as do the vapour pressures of SiO in equilibrium with Si and SiO_2_ and of Si in equilibrium with the Si rich (i.e., the higher) silicides. With alumina forming coatings (e.g., aluminide coatings) the vapour pressure of Al is appreciable at higher temperatures. 

The motivation for the research presented in this paper was the need to develop coating system(s) for Nb-silicide based alloys, in particular to discover αAl_2_O_3_ forming alloys chemically compatible with Nb-silicide based substrates that could be suitable for a multi-material bond coat [3] and/or environmental coatings. Recently, the microstructures and isothermal oxidation of the intermetallic alloys Nb_1.7_Si_2.4_Ti_2.4_Al_3_Hf_0.5_ (alloy MG2) and Nb_1.3_Si_2.4_Ti_2.4_Al_3.5_Hf_0.4_ (alloy MG7) was discussed in [3]. The design of these alloys was guided by the alloy design methodology NICE [27] which used the criteria (i) vol.% Nb_ss_ = 0 and (ii) weight gain per unit area ΔW/A = 0 for isothermal oxidation at 800 and 1200 °C to calculate the concentrations of Si, Ti and Hf. The concentrations of Al were chosen to ensure that specific silicides and aluminides were formed in the microstructures of these alloys. The choice of these intermetallic compounds was discussed in [3]. Tetragonal and hexagonal Nb_5_Si_3_ and only hexagonal Nb_5_Si_3_ were present in the cast microstructures respectively of the alloys MG2 and MG7. Both these alloys exhibited severe macrosegregation of Si and Al, particularly the latter alloy that formed a “layered” microstructure. Both these alloys did not pest and formed alumina in their scales. At 1200 °C the former alloy suffered from internal oxidation and formed a “layered” scale in which the layer that was in contact with the substrate consisted of titanium oxide dispersed in alumina. The alloy MG7 formed a thin continuous well adhering alumina scale that was able to repair itself (self-heal) during oxidation [3]. 

The research presented in this paper is also aimed at extending further the study of alloys of the Nb-Ti-Si-Al-Hf quinary system with objective to “define” a compositional area (map) for alloys of the aforementioned system that can form continuous and self-healing αAl_2_O_3_ in isothermal oxidation in air. In this paper, we report for the first time our research on two new alloys of this system that were designed to form αAl_2_O_3_ scale. The alloys were studied in the as cast and heat treated conditions, and after isothermal oxidation at 800 and 1200 °C, but not as coatings applied on a Nb-silicide based substrate in order to eliminate the effects of substrate and coating process on microstructures and isothermal oxidation (see above). 

The structure of the paper is as follows. First, we discuss how the alloy compositions were selected. This section is followed by a brief description of the experimental techniques that we used. The results for the cast and heat treated microstructures of the alloys and their isothermal oxidation at 800 and 1200 °C are then presented. In the discussion we deliberate on the microstructures of the alloys and the chemical compositions of the intermetallic compounds before we consider the oxidation of the alloys. The latter are then compared with high entropy alloys (HEAs) and the question as to whether the formation of “layered” structures is possible is addressed in the following section where selection of bond coat alloys is discussed. Suggestions for future work are given before the conclusions 

## 2. Design and Selection of the Alloys

The alloy MG2 could not form a continuous thin αAl_2_O_3_ scale and suffered from internal oxidation [3]. The oxides of Hf, Nb and Ti do not form as compact layer during oxidation and therefore permit gaseous transport of oxygen to the substrate. The design and selection of new alloys of the Nb-Ti-Si-Al-Hf system had as objects to eliminate the intermixed Al_2_O_3_/TiO_2_ scale, to decrease the growth of niobates [27] and to form stable αAl_2_O_3_ scale. The design and selection of the two alloys that are the subject of this paper was guided by our understanding of the alloying behaviour of Nb_5_Si_3_ and how it affects its properties [7], the alloy design methodology NICE [27], the research that was reported in [3], the Wagner–Hauffe (W-H) rule [28,29,30,31] and Wagner’s oxidation theory [32]. 

Our study of the alloys MG2 and MG7 [3] would suggest that if the formation of TiO_2_ were suppressed or minimised in alloys of the Nb-Ti-Si-Al-Hf system, then the formation of Al_2_O_3_ rich or an exclusive Al_2_O_3_ scale would be favourable. Diffusion of oxygen via oxygen vacancies will contribute to the growth of TiO_2_. Therefore, alloying elements that can decrease the oxygen vacancies in TiO_2_ will be effective to decrease the overall oxide scale growth rate by inhibiting the fast growth of TiO_2_. In accordance with the W-H rule (also known as the “valence control” rule), addition of Nb which forms cations with a valence greater than +4 will be effective. The suppression of internal oxidation of Al would also be effective to produce a continuous αAl_2_O_3_ scale. Following Wagner, the transition from internal to external oxidation would be enhanced via increasing the diffusion of Al or decreasing the inward diffusion of oxygen. The synergy of Nb with Al and Hf significantly reduces the diffusion of oxygen in ternary Nb-Al-Hf alloys [33].

**Figure 1:**
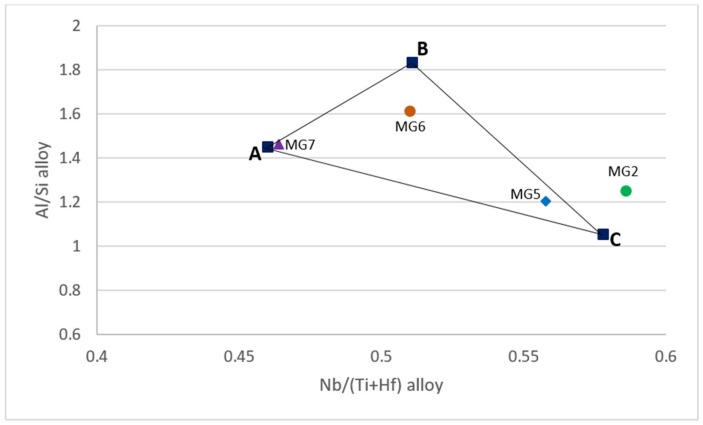
(Al/Si)_alloy_ versus [Nb/(Ti + Hf)]_alloy_ map for Nb-Ti-Si-Al-Hf alloys predicted by NICE [27] to have hexagonal γNb_5_Si_3_ and no solid solution and weight gains per unit area less than or equal to 1 and 10 mg/cm^2^ after 100 h isothermal oxidation respectively at 800 and 1200 °C. The parameters of the alloys Nb_1.7_Si_2.4_Ti_2.4_Al_3_Hf_0.5_ (MG2 [3]), Nb_1.45_Si_2.7_Ti_2.25_Al_3.25_Hf_0.35_ (MG5), Nb_1.35_Si_2.3_Ti_2.3_Al_3.7_Hf_0.35_ (MG6) and Nb_1.3_Si_2.4_Ti_2.4_Al_3.5_Hf_0.4_ (MG7 [3]) are shown for their nominal compositions.

**Figure 2:**
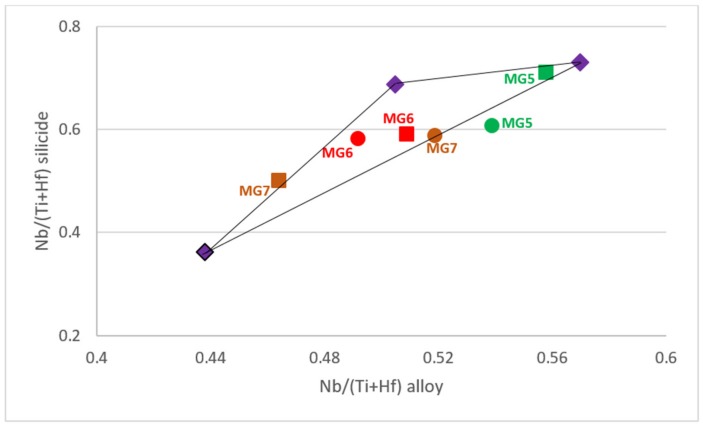
[Nb/(Ti + Hf)]_Nb5Si3_ versus [Nb/(Ti + Hf)]_alloy_ map for Nb-Ti-Si-Al-Hf alloys that are predicted by NICE [27] to form hexagonal γNb_5_Si_3_, have no Nb_ss_ and weight gains per unit area less than or equal to 1 and 10 mg/cm^2^ after 100 h isothermal oxidation respectively at 800 and 1200 °C. Squares show the values corresponding to the nominal compositions and the circles show the actual compositions in all parts of the alloys Nb_1.45_Si_2.7_Ti_2.25_Al_3.25_Hf_0.35_ (MG5), Nb_1.35_Si_2.3_Ti_2.3_Al_3.7_Hf_0.35_ (MG6) and Nb_1.3_Si_2.4_Ti_2.4_Al_3.5_Hf_0.4_ (MG7 [3]).

For Nb_5_Si_3_ and Ti rich Nb_5_Si_3_ the alloy design methodology NICE has relationships that link the concentrations of Al and Si in the silicide with that of Ti, i.e., relationships of the form Al = f_1_(Ti) and Si = f_2_(Ti). There are also relationships between VEC_alloy_ and VEC_Nb5Si3_ is the and Δχ_alloy_ and Δχ_Nb5Si3_ and for the concentration of Ti in Nb_5_Si_3_ which is a function of Δχ_Nb5Si3_ (the parameters Δχ and VEC are related respectively to electronegativity and number of valence electrons per atom filled into the valence band). Figure 1 shows a (Al/Si)_alloy_ versus Nb/(Ti + Hf)_alloy_ map (triangle ABC) constructed using the alloy methodology NICE [27] for intermetallic alloys of the Nb-Si-Ti-Al-Hf system that (a) have no solid solution, (b) form only hexagonal Nb_5_Si_3_ (meaning for alloy and Nb_5_Si_3_ the ratio Nb/(Ti + Hf) is less than one) and (c) have ΔW/A less than 1 and 10 mg/cm^2^ for isothermal oxidation at 800 °C and 1200 °C, respectively. Figure 2 is a [Nb/(Ti + Hf)]_Nb5Si3_ versus [Nb/(Ti + Hf)]_alloy_ map constructed using the alloy methodology NICE [27] for intermetallic alloys of the Nb-Si-Ti-Al-Hf system in which hexagonal Nb_5_Si_3_ is stable and solid solution is not stable.

The two new alloys were designed (i) not to pest, (ii) to form alumina scale at 1200 °C, (iii) to have no solid solution, (iv) to form only hexagonal Nb_5_Si_3_ and (v) to have microstructures consisting of hexagonal Nb_5_Si_3_, Ti_5_Si_3_, Ti_5_Si_4_, TiSi silicides, and tri-aluminides and Al rich TiAl. The reader should refer to [3] where the choice of these intermetallic compounds was discussed.

The two new alloys were chosen to have the same concentration of Hf, namely 3.5 at.%, and to be located inside the (Al/Si)_alloy_ versus [Nb/(Ti + Hf)]_alloy_ and [Nb/(Ti + Hf)]_Nb5Si3_ versus [Nb/(Ti + Hf)]_alloy_ maps for Nb-Ti-Si-Al-Hf alloys (Figure 1 and Figure 2). Furthermore, the compositions of the alloys were adjusted so that (a) their (Al/Si)_alloy_ and Nb/(Ti + Hf)_alloy_ ratios were lower and higher than those of the actual composition of the alloy MG7, (b) they were not as heavily chemically inhomogeneous (macrosegregated) as the alloy MG7 was and (c) their Al concentration was higher or lower than that of the alloy MG7, which might be close to C_kin_^Al^, i.e., the Al concentration that is required for kinetic reasons to form a continuous protective αAl_2_O_3_ scale, see reference [3]. 

The nominal compositions (at.%) of the two alloys of this study were 14.5Nb-27Si-22.5Ti-32.5Al-3.5Hf or Nb_1.45_Si_2.7_Ti_2.25_Al_3.25_Hf_0.35_ (alloy MG5) and 13.5Nb-23Si-23Ti-37Al-3.5Hf or Nb_1.35_Si_2.3_Ti_2.3_Al_3.7_Hf_0.35_ (alloy MG6). The (Al/Si)_alloy_ and Nb/(Ti + Hf)_alloy_ ratios of the alloys MG5 and MG6 respectively were 1.2 and 0.558, and 1.6 and 0.51, compared with 1.5 and 0.526 for the alloy MG7. 

The nominal compositions of the alloys MG5, MG6, MG7 and MG2 are shown in the Figure 1. In the latter, the alloy MG5 is close to corner C, the alloy MG6 is inside the triangle and the alloy MG7 is very close to the corner A. The alloy MG2, in which both tetragonal and hexagonal Nb_5_Si_3_ were formed [3], lies outside the triangle ABC in Figure 1. In the Figure 2 the average values of [Nb/(Ti + Hf)]_Nb5Si3_ and [Nb/(Ti + Hf)]_alloy_ for the actual compositions in all parts of the alloys MG5, MG6 and MG7 are also shown together with the nominal compositions. Note the significant shift in the position of the alloy MG7 in this map owing to the strong chemical inhomogeneity of the alloy [3]. Both alloys satisfy the “standard definition” of high entropy alloys (HEAs) (“alloys with the concentration of each element being between 35 and 5 at.%”) and that both alloys could be considered to be multi-principle element alloys (MPEAs) or complex concentrated alloys (CCAs). 

## 3. Experimental

The two alloys were prepared as small (25 g) buttons by arc-melting high purity (better than 99.9 wt.%) elements in a water cooled copper crucible in an argon atmosphere using a non-consumable tungsten electrode. The melting procedure was repeated 5 times for each alloy. The samples for heat treatment were wrapped in Ta foil and placed in an alumina boat in the centre of a tube furnace. Heat treatments were carried out at 800 and 1200 °C for 100 h in flowing Ti gettered argon. 

Cubic specimens for isothermal oxidation (approximately 3 mm x 3 mm x 3 mm) were cut from the cast buttons. The specimens were polished to 300 grit. Isothermal oxidation experiments were performed at 800 and 1200 °C for 100 h using a NETZSCH STA 49 F3 Jupiter thermal analyser (NETZSCH Gmbh, Seld, Germany) supported by the NETZSCH Proteus software. A Jeol 6400 scanning electron microscope (SEM, Jeol, Tokyo, Japan) and a Philips XL 30S FEG SEM (Philips-ThermoFisher Scientific, Hillsboro, OR, USA) were used for imaging and quantitative analysis. Both instruments were equipped with EDS detectors (Oxford Instruments, Abingdon, UK) and Oxford Instrumentals INCA software for quantitative chemical analysis, and elemental standards of Nb, Ti, Al, Si, Hf. The Philips XL 30S FEG SEM was also equipped with Fe2O3 as the standard for oxygen. The X-ray maps of scales were taken in the latter instrument. All compositions in this paper are given in at.% unless stated otherwise.

A Siemens D5000 diffractometer (Hiltonbrooks Ltd, Crew, UK) with CuKα radiation was used for the identification of phases in the cast and heat treated specimen and for glancing angle XRD (GXRD) to identify the oxides in the scales that formed on the oxidised specimens. The GRXD was performed at a scan speed of 2 degrees per minute over a 2θ range of 20 to 100 with glancing angle of 5 degrees. For phase analysis the ICDD (International Centre for Diffraction Data) PDF-4+ database and SIeve+ software (ICDD, Newton Square, PA, USA) was used.

## 4. Results

### 4.1. Cast Alloys

The actual compositions of the alloys MG5 and MG6 respectively were Nb-24.2Ti-26.7Si-30.6Al-3.4Hf and Nb-22.7Ti-23.3Si-37.7Al-3.6Hf. These were the average compositions of all the analyses taken from the top, bulk and bottom of each button. Even though the alloys were close to their nominal compositions, particularly the alloy MG6, both alloys were chemically inhomogeneous (Figure S1 in the Supplemental data) with significant standard deviations for Al, Si and Ti (in decreasing order) that were higher for the alloy MG5. In the latter alloy the difference between maximum and minimum concentration of these elements was 21.9 at.% for Al, and 9.9 at.% for Si with the strongest inhomogeneity of Al and Si in the bulk, and 7 at.% for Ti with the strongest inhomogeneity in the bottom of the button. In the alloy MG6 the minimum and maximum concentration of Al, Si and Ti were 30.7 and 49.8 at.% Al, 16.5 and 27.3 at.% Si and 20.7 and 24.7 at.% Ti, with the strongest inhomogeneity for all these elements in the bulk of the button.

**Figure 3:**
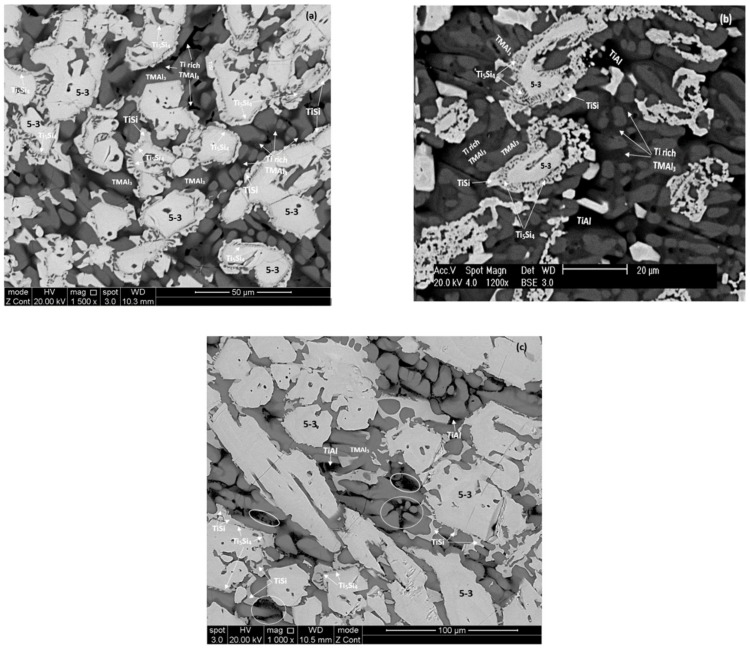
Back scatter electron (BSE) images of the microstructures of the cast alloys (**a**) top—MG5, (**b**) and (**c**) respectively bottom and bulk of the alloy MG6. For the encircled areas see text.

The phases that were identified by XRD and quantitative EDS analysis are summarised in Table 1. The XRD data for the cast alloys is shown in Figure S2 in the Supplemental data. Figure 3 shows the microstructures of the cast alloys. In all parts of the button the microstructure of the alloy MG5 consisted of hexagonal γNb_5_Si_3_ (identified as 5-3 in Figure 3a), Ti_5_Si_4_, TiSi and TMAl_3_ (TM is transition metal). Depending on its Ti content the tri-aluminide exhibited light, dark and very dark contrast (identified as Ti rich TMAl_3_ in Figure 3a). Its average composition was 10.6(±3.5)Nb-3.2(±0.7)Si-14.4(±3.4)Ti-70.7(±1.3)Al-1.1(±0.2)Hf, where in parenthesis is given the standard deviation. The γNb_5_Si_3_ was surrounded by Ti_5_Si_4_, which was surrounded by TiSi (“composite” silicide) and the TMAl_3_ formed in-between the “composite” silicide grains. In the bulk of the button few large grains were Ti_5_Si_4_ surrounded by TiSi. The volume fractions of γNb_5_Si_3_ and Ti_5_Si_4_ were higher in the bulk of the button compared with the top and bottom. The microstructure in the part of the latter that was in contact with the water cooled copper crucible was finer compared with the bulk and top of the button. The fibrous (wool like) structure of Ti_5_Si_4_ [3] was more pronounced in the top of the button. No layered structure was observed in the cast alloy MG5. 

The same phases plus TiAl were observed in all parts of the button of the alloy MG6 (Table 1). The microstructure in the bottom of the button consisted of hexagonal γNb_5_Si_3_ (identified as 5-3 in Figure 3b), Ti_5_Si_4_, TiSi, TiAl and TMAl_3_. There was strong partitioning of Ti in the tri-aluminide that exhibited light, dark and very dark contrast (identified as Ti rich TMAl_3_ in Figure 3b). Its average composition was 8.1(±6.7)Nb-4.4(±3)Si-16.4(±6.1)Ti-70.3(±2.5)Al-0.8Hf, where in parenthesis is given the standard deviation. Ti rich TiAl formed in-between tri-aluminide grains and exhibited very dark contrast (average composition 2.1Nb-6.2Si-36.5Ti-54.2Al-1Hf). It was not possible to distinguish the TMAl_3_ and TiAl only on the basis of their contrast under BSE imaging, owing to the partitioning of Ti in both these intermetallic compounds. In the bulk and top of the button the Ti_5_Si_4_ and TiSi compounds and in particular the fibrous structure of the former were not as well developed compared with the bottom of the button (compare Figure 3b,c). Furthermore, in some of the areas that exhibited a very dark contrast there was evidence of very fine structure, most likely a eutectic. Some of these areas are indicated by circles in Figure 3c.

**Figure 4:**
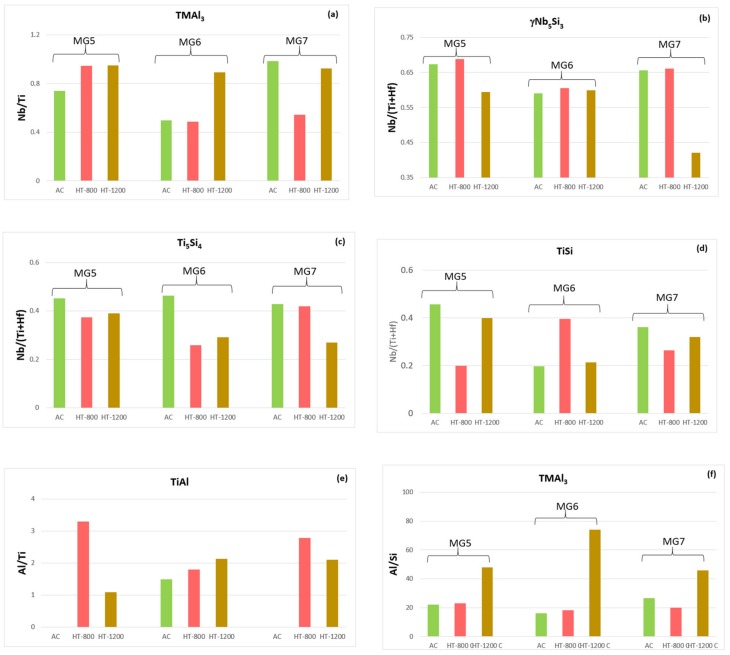
Data about the ratios Nb/Ti, Nb/(Ti + Hf), Al/Ti and Al/Si for different intermetallic compounds observed in the cast and heat treated alloys MG5, MG6 (this study) and MG7 [3], (**a**) TMAl_3_, (**b**) γNb_5_Si_3_, (**c**) Ti_5_Si_4_, (**d**) TiSi, (**e**) TiAl, (**f**) TMAl_3._

Figure S3a–d in the Supplemental data respectively show the average concentrations of Si, Ti, Al, Nb and Hf in the TMAl_3_, γNb_5_Si_3_, Ti_5_Si_4_ and TiSi intermetallic compounds in the alloys MG5 and MG6. In the former three intermetallic compounds the average concentrations of these elements were essentially similar in the two alloys but their standard deviations were significantly higher in the alloy MG6. In the latter, the TMAl_3_ and γNb_5_Si_3_ compounds had lower Nb/Ti and Nb/(Ti + Hf) ratios compared with the alloy MG5 but the Nb/(Ti + Hf) ratio of Ti_5_Si_4_ was slightly higher (Figure 4a–c). Compared with the alloy MG5, the TiSi compound was richer in Si and Ti and poorer in Al and Nb in the alloy MG6 and its Nb/(Ti + Hf) ratio was lower (Figure 4d). The TiAl, which was observed only in the alloy MG6, had Al/Ti = 1.485 (Figure 4e). The tri-aluminide in the alloy MG6 was richer in Si compared with the alloy MG5 (Figure 4f).

### 4.2. Heat Treated Alloys

#### 4.2.1. Heat Treatment at 800 °C

The average compositions were Nb-22.6Ti-24.7Si-26.8Al-3.3Hf and Nb-22.9Ti-23.6Si-36.8Al-3.7Hf for the heat treated specimens of the alloys MG5 and MG6, respectively. Chemical inhomogeneity persisted in both alloys, especially in the alloy MG5 (Figure S1 in the Supplemental data), in which the standard deviations of Ti, Si and Al (in increasing order) were higher than those in the alloy MG6, particularly for Al.

**Figure 5:**
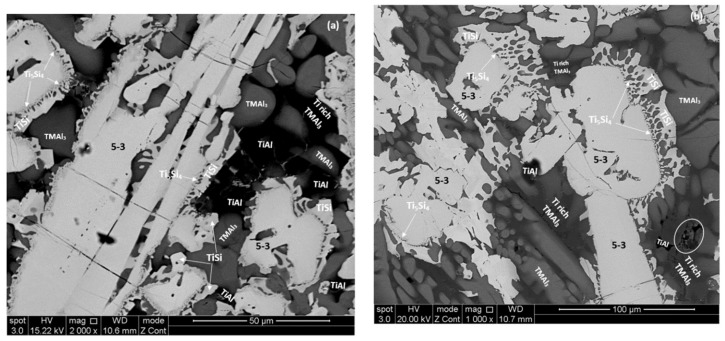
BSE images of the microstructures of the heat treated alloys at 800 °C (**a**) MG5, (**b**) MG6.

The phases that were identified by XRD and quantitative EDS analysis are summarised in Table 1. The XRD data for the alloys is shown in Figure S4 in the Supplemental data. In both alloys the TiAl was observed in-between TMAl_3_ areas and in both phases there was variation in Ti concentration which had a strong effect on their contrast under BSE imaging, thus making their identification on the basis of contrast impossible. In Ti rich areas in-between the TiAl and TMAl_3_ confined by the large “composite” silicide grains the Ti rich TM_9_Si_7_Al_4_ intermetallic was also observed in the alloy MG6. The average composition of this phase was 10.7(±3.8)Nb-30.3(±1.6)Ti-35(±2.6)Si-20.2(±5.5)Al-3.8(±0.3)Hf. Figure 5 shows the microstructures of the heat treated alloys. Prior “eutectic” in the microstructure of MG6 is shown by the circle in the bottom right hand corner of the Figure 5b.

The average compositions of the intermetallic phases that were observed in both alloys are compared in the Figure S3 in the Supplemental data. Even though the TMAl_3_ and γNb_5_Si_3_ had essentially similar composition in each alloy (Figure S3a,b in the Supplemental data), the Nb/Ti ratio of the former had increased in MG5 and did not change in MG6 (Figure 4a) and the Nb/(Ti + Hf) ratio of the latter had increased in both alloys but was still lower (0.605) in MG6 (Figure 4b). The Ti_5_Si_4_ was richer in Ti and Al in MG6 (Figure S3c in the Supplemental data) and its Nb/(Ti + Hf) ratio had decreased more (Figure 4c). The composition of TiSi was different in the two alloys (Figure S3d in the Supplemental data), this compound was richer in Al and Ti in the alloy MG5 and had a lower Nb/(Ti + Hf) ratio compared with the alloy MG6 (Figure 4d). The TiAl was richer in Al and poorer in Si and Ti in the alloy MG6 (Figure S3e in the Supplemental data) and had a lower Al/Ti ratio compared with the alloy MG5 (Figure 4e). The Si content of the tri-aluminide did not change significantly in both alloys (Figure 4f).

#### 4.2.2. Heat Treatment at 1200 °C

The average compositions of the heat treated specimens of the alloys MG5 and MG6 respectively were Nb-24.1Ti-26Si-31.5Al-3.7Hf and Nb-23Ti-23.2Si-37.1Al-3.5Hf. Chemical inhomogeneity persisted in both alloys, especially in the alloy MG5 (Figure S1 in the Supplemental data), in which the standard deviations of Ti, Si and Al (in increasing order) were higher than those in the alloy MG6, particularly for Al. The strongest chemical inhomogeneity was observed in the bulk of the alloy MG5. 

**Figure 6:**
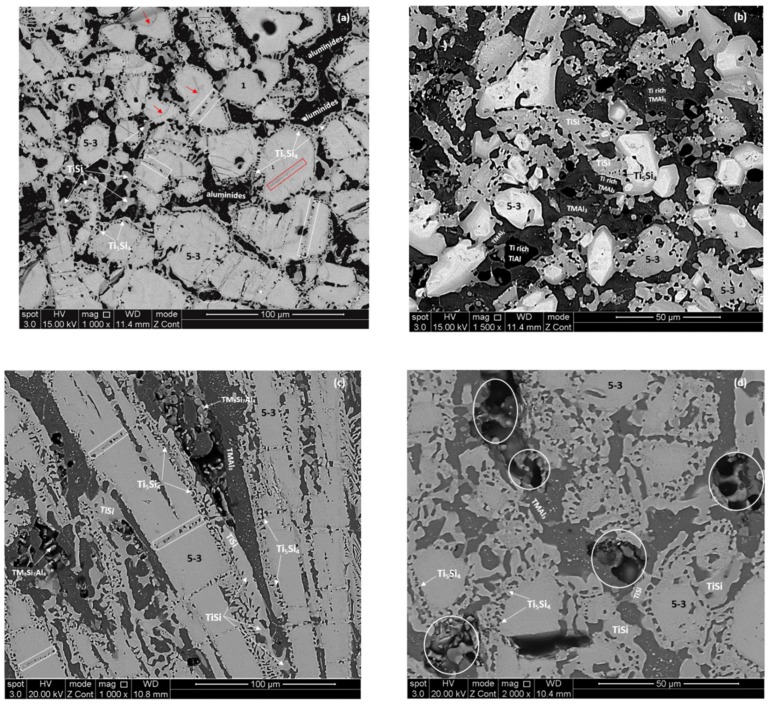
BSE images of the microstructures of the heat treated alloys at 1200 °C (**a**) and (**b**) MG5, (**c**) and (**d**) MG6. In (b) the contrast of the image has been enhanced in order to show the different contrasts of aluminides formed between the γNb_5_Si_3_ grains. In (d) the circles indicate areas where incipient melting had occurred.

The phases that were identified by XRD and quantitative EDS analysis are summarised in Table 1. The XRD data for the alloys is shown in Figure S5 in the Supplemental data. The same phases were observed in both alloys, namely γNb_5_Si_3_, TMAl_3_, Ti_5_Si_4_, TiSi and TiAl. Note that the Ti rich TM_9_Si_7_Al_4_ intermetallic was not observed by XRD in the alloy MG6, as was the case at 800 °C. The microstructures of the alloys are shown in Figure 6. The alloy MG6 exhibited incipient melting in areas in-between some “composite” silicide grains (Figure 6d).

In the alloy MG5 the majority of the γNb_5_Si_3_ grains (i) were cracked, with cracks running parallel to each other and sometimes crossing from one grain to a neighbouring grain (Figure 6a), (ii) were “partitioned” by microstructures that were similar to those of Ti_5_Si_4_ (indicated by white rectangles in Figure 6a), and (iii) exhibited “lines” of darker contrast that were either “straight” (indicated by red rectangle in Figure 6a) or curved (indicated by red arrows in Figure 6a). A few γNb_5_Si_3_ grains were free of (i) to (iii), for example see grain 1 in the top right hand side of Figure 6a, while in some γNb_5_Si_3_ grains there was evidence of fine second phase(s) in areas of grey and bright contrast, for example see grain 1 in the bottom right hand side of Figure 6b. Aluminides were present in-between the large “composite” silicide grains (Figure 6a) that were identified as TMAl_3_ and TiAl with strong partitioning of Ti in both these compounds (indicated as Ti rich TMAl_3_ and Ti rich TiAl in Figure 6b). There was also evidence of fine second phase(s) forming in the area of aluminides (see encircled area in the bottom of the Figure 6a). The fine phases were formed in TMAl_3_, see Figure 6b. There was no evidence of incipient melting in the alloy MG5. 

In the alloy MG6 there was “partitioning” of γNb_5_Si_3_ grains by microstructures that were similar to those observed in the alloy MG5 (indicated by white rectangles in Figure 6c) but we did not observe (i) the severe cracking of γNb_5_Si_3_ grains and (ii) the darker contrast “straight” or curved lines in γNb_5_Si_3_ grains, and we did not find evidence of fine second phase(s) in γNb_5_Si_3_ grains. There was evidence of incipient melting in some areas where aluminides were observed in the cast and heat treated at 800 °C microstructures (see encircled areas in Figure 6d) and also evidence of fine second phase(s) forming in TMAl_3_ (Figure 6c,d).

The average compositions and the Nb/Ti and Nb/(Ti + Hf) ratios respectively of TMAl_3_ and γNb_5_Si_3_ in the alloys MG5 and MG6 essentially were similar Figure 4a,b) but the standard deviations for Al and Si for the γNb_5_Si_3_ in the latter alloy were higher (21.4 (±1.8)Nb-30.4(±1.2)Ti-37.3(±1.5)Si-5.4(±2.9)Al-5.4(±0.6)Hf). The Ti_5_Si_4_ was slightly richer in Ti in the alloy MG6 and had a lower Nb/(Ti + Hf) ratio compared with the alloy MG5 (Figure 4c). In the alloy MG6 the TiSi was richer in Al and Ti and poorer in Nb (8.9(±1.1)Nb-35.9(±5.9)Si-37.7(±4.)Ti-13.3(±4.2)Al-4.1(±1.3)Hf) and like Ti_5_Si_4_ had lower Nb/(Ti+Hf) ratio than in the alloy MG5 (Figure 4d). The TiAl was richer in Ti and poorer in Si and Nb and had a lower Al/Ti ratio in the alloy MG5 (3.8(±0.9)Nb-7.3(±1)Si-41.8(±3.9)Ti-45.2(±3)Al-1.8(±1.2)Hf) compared with the alloy MG6 (Figure 4e). The Si content of the tri-aluminide decreased significantly in both alloys (Figure 4f). The average composition of the TM_9_Si_7_Al_4_ intermetallic was similar to that after the heat treatment at 800 °C (see previous section).

### 4.3. Isothermal Oxidation

**Figure 7:**
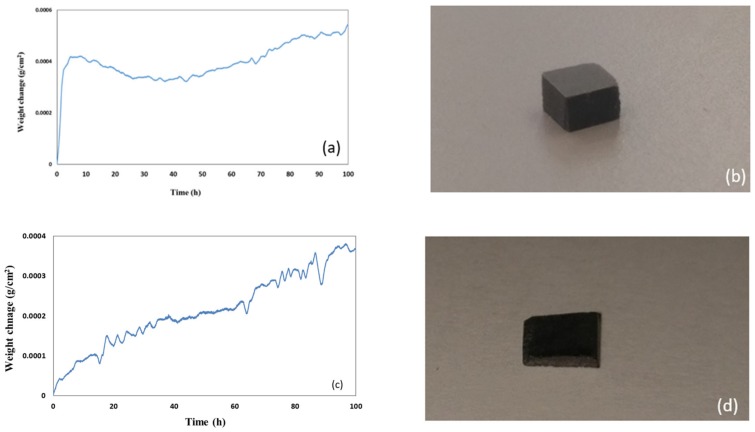
Weight change data and oxidised specimens after isothermal oxidation at 800 °C (**a**,**b**) alloy MG5, (**c**,**d**) alloy MG6.

**Figure 8:**
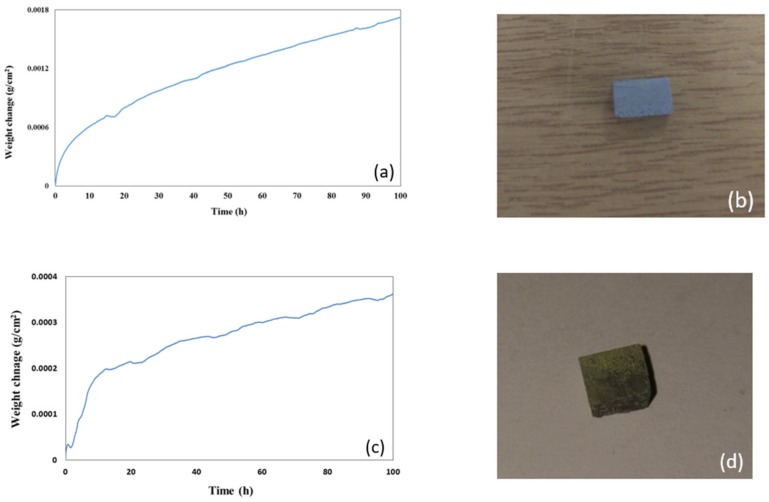
Weight change data and oxidised specimens after isothermal oxidation at 1200 °C (**a**,**b**) alloy MG5, (**c**,**d**) alloy MG6.

The data about weight change per unit area and the oxidised specimens after oxidation at 800 and 1200 °C respectively are shown in Figure 7 and Figure 8. Both alloys did not pest at 800 °C. The alloy MG5 lost weight in the early stages (< 25 h) of oxidation at 800 °C, then its weight changed very little up to about 42 h and after this time it gained a small weight (overall weight change 0.5 mg/cm^2^). At the same temperature, the alloy MG6 gained 0.37 mg/cm^2^. At 1200 °C both alloys followed parabolic oxidation kinetics, the alloy MG5 gained 1.6 mg/cm^2^ and had k_p_ = 8 × 10^−12^ g^2^/cm^4^s (R^2^ = 0.9994) and the alloy MG6 gained 2.3 mg/cm^2^ and had k_p_ = 3 × 10^−13^ g^2^/cm^4^s (R^2^ = 0.97).

**Figure 9:**
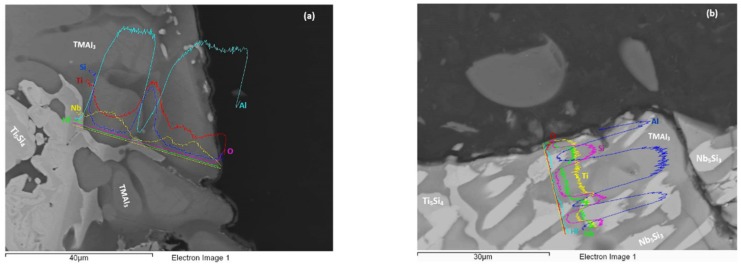
BSE images and EDS line scans from scale to bulk of oxidised specimens at 800 °C of the alloys MG5 (**a**) and MG6 (**b**).

**Figure 10:**
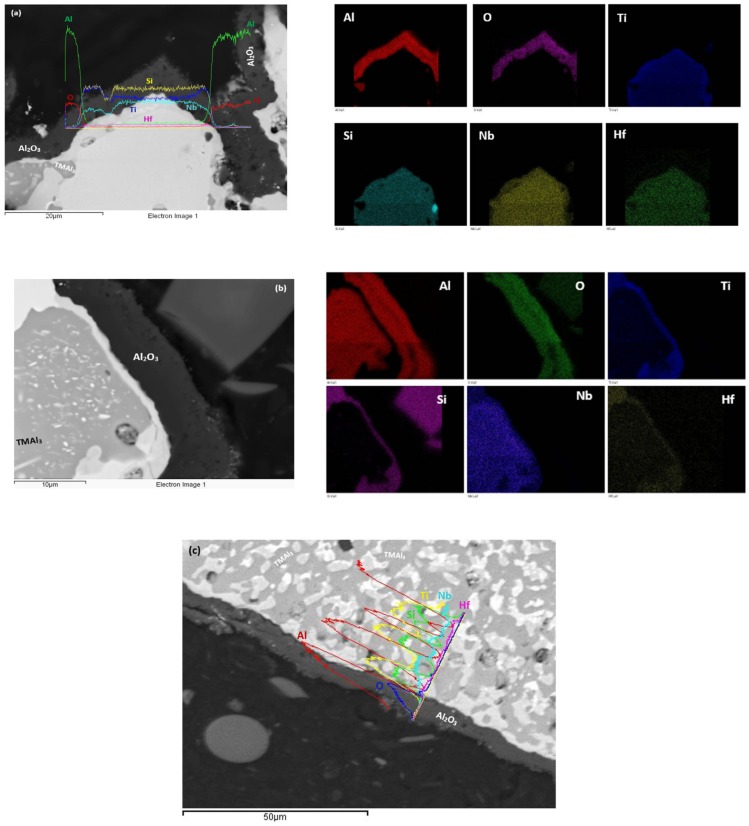
(**a**,**b**) BSE images and corresponding elemental maps of oxidised specimens at 1200 °C of the alloy MG5 and (**c**) EDS line scans from scale to bulk of oxidised specimen of the alloy MG6 at 1200 °C.

At each temperature both the alloys formed thin scales. At 800 °C the scales were thinner (≤2 μm) compared with those at 1200 °C (≤10 μm). Furthermore, the scales of the alloy MG5 were slightly thinner than those of the alloy MG6. The scales that formed at 800 °C are shown in Figure 9. The scale of the alloy MG5 was continuous and adhered well to the substrate. The line scans in Figure 9 show that both scales were rich in Al. Both alloys formed thicker continuous Al_2_O_3_ scales at 1200 °C (Figure 10). Alumina scale formed on top of silicides and aluminides (Figure 10). 

The GXRD data for the oxidised specimens after oxidation at 800 and 1200 °C respectively is shown in Figures S6 and S7 in the Supplemental data. For both alloys the GXRD confirmed the presence of αAl_2_O_3_ at 1200 °C (Figure S7 in the Supplemental data) and TiAl_2_O_5_ at 800 and 1200 °C (Figures S6 and S7 in the Supplemental data). For both temperatures the GXRD suggested the presence of Ti niobates and hafnia, and SiO_2_ at 1200 °C. The latter oxide was also suggested by GXRD for the alloy MG6 at 800 °C. Evidence for Ti containing oxide(s) can be seen in the top right hand side of Figure 10a and top right hand side of Figure 10b (light blue contrast).

The microstructures in the bulk of the oxidised specimens were the same as those of the heat treated specimens at 800 and 1200 °C (see Table 1, Figure 5 and Figure 6). Just below the alloy/scale interface TMAl_2_ with Al + Si ≈ 67 at.% (10.6Nb-5.1Si-21.2Ti-62.1Al-0.9Hf) was observed together with TMAl_3_ in both alloys and at both temperatures. The presence of TiAl_2_ was confirmed by XRD (data not shown) at both temperatures and for both alloys. Furthermore, the EDS data taken from the alloy/scale interface indicated the presence of Ti_2_Al_5_ with Al + Si ≈ 71.3 at% (12.7Nb-1.5Si-15.3Ti-69.8Al-0.6Hf), TM_2_Si_1.5_Al (14.3Nb-31.2Si-27.2Ti-23Al-4.3Hf) in the alloy MG5 at both temperatures and TM_8_Al_11_Si_3_ (9.2Nb-14.8Si-20.3Ti-54Al-1.6Hf) only at 800 °C and in the case of the alloy MG6 the EDS indicated the presence of TM_2.3_AlSi_0.3_ (2.6Nb-7.5Si-64Ti-24.7Al-1.1Hf) at 1200 °C.

## 5. Discussion

### 5.1. Macrosegregation

The microstructures of both the cast alloys were chemically inhomogeneous. The macro-segregations of Si (MACSi), Al (MACAl) and Ti (MACTi) were less severe compared with the alloy MG7 [3] (the macrosegregation of element i is defined as the difference between the maximum and minimum values of the element i, i.e., MACi = C_max_^i^ – C_min_^i^ [34]). The chemical inhomogeneity of the above elements persisted after the heat treatment as was the case in the alloy MG7. Compared with the alloy MG1 [35], the MACSi increased as the parameters T_m_^alloy^, ΔH_m_^alloy^, ΔH_m_^sd^, T_m_^sd^ decreased and the parameter T_m_^sp^ increased, in agreement with [34] (see [34] for the definition of parameters).

### 5.2. Microstructures

#### 5.2.1. Cast Alloys

In each alloy the same phases were observed in all parts of the button and in both alloys the hexagonal γNb_5_Si_3_ was the primary phase in agreement with the Ti-Al-Si system [36] when both alloys are considered as (Ti,Nb,Hf)-Si-Al alloys. In both alloys “composite” silicide grains were formed that consisted of γNb_5_Si_3_ core “surrounded” by Ti_5_Si_4_ which was “surrounded” by TiSi. In the alloy MG6 the TiAl aluminide was formed (Table 1) and there was evidence of a eutectic that included silicide(s) and aluminide(s) (Figure 3c). The aluminide(s) was (were) formed in between the “composite” silicide grains. Some “composite” grains that consisted only of Ti_5_Si_4_ surrounded by TiSi (meaning there was no γNb_5_Si_3_ “core”) were observed in the bulk of the alloy MG5. This was attributed to higher Si concentration in the melt in these areas (see Figure S1 in the Supplemental data).

The solidification path of the alloy MG5 was the same as that of the alloy MG7 [3], namely L → L + γNb_5_Si_3_ → L + γNb_5_Si_3_ + TM_5_Si_4_ → L + γNb_5_Si_3_ + TM_5_Si_4_ + TiSi → γNb_5_Si_3_ + TM_5_Si_4_ + TiSi + TMAl_3_. The solidification path of the alloy MG6 was L → L + γNb_5_Si_3_ → L + γNb_5_Si_3_ + TM_5_Si_4_ → L + γNb_5_Si_3_ + TM_5_Si_4_ + TiSi → L + γNb_5_Si_3_ + TM_5_Si_4_ + TiSi + TiAl + TMAl_3_ → γNb_5_Si_3_ + TM_5_Si_4_ + TiSi + TiAl + TMAl_3_ + eutectic. In both alloys the aluminide(s) formed from Si containing melts. The Si concentration of melts in equilibrium with TiAl_3_ varies slightly with temperature [37]. In both alloys the formation of aluminide(s) was “controlled” by the Ti-Al-Si phase equilibria and that of TMSi by the Nb-Ti-Si phase equilibria [38]. The same was the case in the alloy MG7 [3].

#### 5.2.2. Heat Treated Alloys

The TiAl and the TM_9_Si_7_Al_4_ were the new intermetallic compounds observed in the heat treated microstructures at 800 and 1200 °C respectively of the alloys MG5 and MG6 (Table 1). It was concluded that the TiAl is stable in both alloys. Its formation can be accounted for by the Ti-Al-Si phase equilibria at 700 °C [39,40] and 1200 °C [39,41]. Equilibrium between Ti_5_Si_4_, Ti_5_Si_3_ and TiAl_3_ at 1100 °C and between Ti_5_Si_4_, TiSi and TiAl_3_ at 700 °C can be accounted by Ti-Al-Si phase equilibria [42,43]. The presence of TM_9_Si_7_Al_4_ was confirmed only by quantitative EDS and cannot be accounted for by the available phase equilibria data. The structure of this compound is not known. It had Al + Si ≈ 55 at.% and Al/Si ≈ 0.6. 

The molar volumes (cm^3^/g-atom) of TiAl_3_, Ti_2_Al_5_, TiAl_2_, TiAl, TiSi, Ti_5_Si_4_ and Ti_5_Si_3_ respectively are 9.598, 9.539, 9.667, 9.813, 8.970, 11.040 and 10.670 [42]. The molar volume of Ti_5_Si_4_ is the highest and of TiSi the lowest. Large volume reduction would accompany the formation of Ti_5_Si_4_ from TiSi and Ti and large volume expansion would accompany the formation of Ti_5_Si_4_ from TiAl_3_ and Si [42]. As the fractions of the silicides changed during the exposure to high temperature in each heat treatment, these changes were accompanied by significant volume changes that gave rise to stresses that resulted to the cracking of the “composite” silicide grains. 

The partitioning of γNb_5_Si_3_ grains by Ti_5_Si_4_ like structure might be linked with the formation of dark contrast straight or curves areas (lines) at 1200 °C (Figure 6). At this temperature the Ti concentration and the Nb/(Ti + Hf) ratio of the γNb_5_Si_3_ respectively increased (Figure S3 in the Supplemental data) and decreased (Figure 4b). The change in contrast could be attributed to the partitioning of Ti in the silicide which induced a reaction that resulted in the formation of Ti_5_Si_4_ and enrichment of the surrounding γNb_5_Si_3_ in Al (owing to the different solubilities of Al in the 5-4 and 5-3 silicides). Thus, the γNb_5_Si_3_ was surrounded by Ti_5_Si_4_ (with Al enrichment at the γNb_5_Si_3_ side of the γNb_5_Si_3_/Ti_5_Si_4_ interface), and the Ti_5_Si_4_ was surrounded by Al rich TiSi (Figure S3d in the Supplemental data), which “protected” the γNb_5_Si_3_ core and improved the oxidation of the “composite” silicide grains at 1200 °C.

The DSC trace for the alloy MG6 (not shown) displayed an exothermic peak around 700 °C, which was accompanied by a small weight gain in the TG trace. This peak could be attributed to the formation of TM_9_Si_7_Al_4_ and/or Ti(Al_1−*x*_Si*_x_*)_3_ (tI8, I4/mmm, TiAl_3_ (h)) with 0 < x < 0.15. The latter compound is stable above 735 °C [44]. The DSC trace of the alloy MG6 also showed an endothermic peak on heating around 1100 °C with an accompanying rapid increase in weight gain in the TG trace. This could be associated with the reaction L + Ti_5_Si_4_ ↔ TiSi + TiAl_3_ at just above 1105 °C [45] and could account for the localised melting observed at 1200 °C (Figure 6d). It should be noted that the composition of the cast alloy MG6, from which the specimen for the DSC study was taken, falls in the three phase Ti_5_Si_4_, TiSi, TiAl_3_ area at 1100 °C in the solidus surface of the Ti-Al-Si system [36]. It is highly likely that localised melting occurred in the areas where eutectic like structure was observed in the cast alloy (Figure 3c). We could not confirm whether the presence of the TM_9_Si_7_Al_4_ compound played a role in the localised melting.

#### 5.2.3. Chemical Composition of Intermetallic Compounds

In both alloys the solubilities of Si and Al respectively in TiAl and Ti_5_Si_4_ were in agreement with [36,42] but the solubility of Al in TiSi was higher than that reported in [42]. In both alloys and for both temperatures the solid solubility of Si in TiAl was higher than the maximum solid solubility reported in [43]. Furthermore, in both alloys the solid solubility of Si in TiAl increased with increasing temperature, in agreement with the data for the alloy MG7 [3]. The solid solubility of Al in Ti_5_Si_4_ was in agreement with [3].

The solubility of Al in the Nb_5_Si_3_ silicide in the alloys MG5 and MG6 (and MG7) was lower than that reported in [43] and the solubilities of Hf and Ti in Nb_5_Si_3_ in the alloys MG5 and MG6 (and MG7) were in agreement with the literature [7,19]. 

Silicon has a strong preference for the Al sublattice in TiAl_3_ with Si ≤ 16–17 at.% [46]. The solid solubility of Si in TMAl_3_ in both the cast and heat treated alloys was in the ranges reported in [37,46,47] but significantly lower than the reported maximum values and decreased with increasing temperature. The solid solubility of Si in TiAl_2_ was higher than that reported in [43] but the Al + Si content of this compound was essentially the same.

#### 5.2.4. Fibrous Ti_5_Si_4_

The fibrous (wool like) structure of the Ti_5_Si_4_ silicide has been reported by Gupta [48], Park et al [42] and Park and Kim [49] in diffusion couples, and was also observed in the as cast and heat treated alloy MG7 [3]. The fibrous structure of the Ti_5_Si_4_ silicide around the γNb_5_Si_3_ silicide was more pronounced (well developed) in the top and bottom of the cast buttons, respectively of the alloys MG5 and MG6, see Figure 3a,b and in the Zone B, bulk and top of the button of the alloy MG7 [3] and in all areas the fibrous structure developed further after the heat treatment. In the cast alloys MG5 and MG6 both areas were richer in Al and poorer in Si and Ti and had the highest (Al + Si)/Ti ratio compared with the other areas of the buttons (Figure S1 in the Supplemental data). In the alloy MG7 in the areas where the fibrous Ti_5_Si_4_ was observed the (Al + Si)/Ti ratio was essentially the same. These observations would suggest (i) that the formation of the fibrous Ti_5_Si_4_ structure depended (a) on the concentration of Al, Si and Ti in the melt that surrounded the “composite” silicide grains and (b) on the fraction of the Ti_5_Si_4_ that formed around the γNb_5_Si_3_ which depended on the Si concentration in the melt surrounding the γNb_5_Si_3_. Park and Kim [49] suggested that Al affects the growth of Ti_5_Si_4_. In the bulk of the alloy MG6 the chemical inhomogeneity of Al was strongest.

### 5.3. Oxidation

Some alloys can form protective oxide at low temperatures and others at high temperatures. The effect of temperature and alloying additions on the selective oxidation of an element is linked with how temperature and alloying element affect oxygen permeability and solute diffusivity in the alloy and the growth rate of transient oxide. In [3] it was discussed that such data is not available for the Nb-Si-Ti-Al-Hf system. 

According to Wagner, the thermodynamic and diffusional properties of the alloy immediately beneath the oxide are important for (determine) the concentration of solute that is required to maintain the growth of an external scale [50]. In the case of intermetallic compounds with narrow or no solubility ranges (in our case the TiSi, Ti_5_Si_4_, Ti_5_Si_3_ and Nb_5_Si_3_ silicides and the TiAl_3_, Ti_2_Al_5_ and TiAl_2_ aluminides) the consumption of the element that forms the external oxide results in the formation of the next intermetallic compound with lower concentration of the consumed element next to the external oxide. The properties of the lower intermetallic compound determine the ability of the intermetallic to maintain the growth of the protective oxide. 

In the case of TiSi the lower compound is Ti_5_Si_4_ and the lower compound of the latter is the hexagonal Ti_5_Si_3_, which has superior oxidation behaviour than Nb_5_Si_3_ that even improves when contaminated by oxygen [51]. Furthermore, the TiSi, Ti_5_Si_4_ and hexagonal Ti_5_Si_3_ do not pest. The “composite” silicide was expected not to pest and to have superior oxidation than that of Nb_5_Si_3_, particularly as it was surrounded by Ti5Si4 with Al enrichment at the interface (see Section 5.2.2). In the case of TiAl_3_ the lower compounds are Ti_2_Al_5_ and TiAl_2_ all of which do not pest and form alumina scale (see [3] for discussion of relevant literature). In the case of NbAl_3_, which is isomorphous with TiAl_3_ but has a narrow solubility range, the lower compound is Nb_2_Al, which has poor oxidation and both aluminides suffer from pest oxidation. However, alloying the NbAl_3_ with Ti promotes external αAl_2_O_3_ scale formation at lower concentrations than those required for NbAl_3_ [52]. In the case of alumina forming Al rich TiAl the lower compound is titania forming Al poor TiAl. Thus, it was essential that the tri-aluminide(s) is(are) Ti rich (Nb/Ti < 1) and the TiAl Al rich (Al/Ti>1). Indeed, this was the case for the aluminides in the alloys MG5 and MG6 (and MG7), see Figure 4.

The γNb_5_Si_3_, Ti_5_Si_4_ and TiSi had Nb/(Ti + Hf) < 1 in all three alloys and this ratio had the lowest value at 1200 °C for the alloys MG5 and MG7 (Figure 4b–d). In the TiSi the trends of the Nb/(Ti + Hf) ratio were the same in all three alloys (Figure 4d). The TMAl_3_ had Nb/Ti < 1 for all three alloys and this ratio was high (the aluminide became poorer in Nb) at 1200 °C (Figure 4a). The Al/Si ratio of the TMAl_3_ was very high for all three alloys and had its highest value (the tri-aluminide became poorer in Si) at 1200 °C (Figure 4f). The TiAl had Al/Ti > 1 in all three alloys, the ratio (i) increased with heat treatment temperature in the alloy MG6, (ii) exhibited the same trend in the alloys MG5 and MG7, (iii) was the same at 1200 °C in the alloys MG6 and MG7 and (iv) had the lowest (but still greater than one) value at 1200 °C for the alloy MG5 (Figure 4e).

The two alloys of this study did not have “a base metal”. The same is the case for the intermetallic compounds observed in their microstructures (Table 1), with the exception of the TiAl aluminide, which was very poor in Nb (i) in the cast alloy MG6 (about 2.1 at.%, see Section 4.1), (ii) in the heat treated alloy MG5 at 800 and 1200 °C, where its Nb content was about 4 at.%, and (iii) in the heat treated alloy MG6 at 800 °C, where the Nb concentration was less than 1 at.%. In other words, it is not easy to indicate which would be the “lower compound” of the majority of the intermetallics in the microstructures of the two oxidised alloys. In addition, in both alloys “composite” silicide grains were formed where the hexagonal γNb_5_Si_3_ silicide core was surrounded by higher not lower compounds. This was also the case for the alloy MG7 [3].

Do the oxidation responses of the two alloys at 800 °C, where they did not pest, and at 1200 °C, where they formed a continuous thin well adhering αAl_2_O_3_ scale with no internal oxidation, point to some form of “cocktail effect” [53] and therefore unexpected synergies between elements and/or intermetallic phases in each alloy? Were the solubility and diffusivity of oxygen and the solute diffusivities in the alloys affected by synergies between elements and/or intermetallic phases? Which (if any) were the synergistic mixtures of elements and/or phases in each alloy at 800 °C? Which were the synergistic mixtures of elements and/or intermetallic compounds that gave the exceptional oxidation of the two alloys. Were these the same as in the case of the alloy MG7? Was the oxidation behaviour of each alloy greater than the sum of constituent parts? Was the oxidation at each temperature determined only by activities and partial pressures of oxygen? Was the oxidation of each alloy some combination of the above? These questions were raised in our previous paper [3] and still remain unanswered. As was the case in [3] we shall discuss the oxidation of the alloys MG5 and MG6 by referring to their starting and/or heat treated microstructures, current knowledge about the oxidation of binary or ternary intermetallic phases and data about the thermal expansion of compounds and oxides.

The starting microstructure of the oxidation specimens of the two alloys are shown in the Table 1. At 800 °C both alloys did not pest, even though at this temperature the γNb_5_Si_3_ was cracked, most severely in the alloy MG5 (Figure 5), and formed thin scales that were rich in Al in both alloys (Figure 9). Weight loss was exhibited only by MG5 and was attributed to Al and Si loss for the same reasons as discussed in [3]. 

Weight loss in isothermal oxidation at 800 °C was also observed for the alloy MG2 but not for the alloy MG7 [3]. The alloys MG2 and MG5 (a) were close the corner C on the triangle ABC in Figure 1, the former outside and the latter inside the triangle, (b) essentially had the same (Al/Si)_alloy_ ratio (1.25 for MG2 and 1.2 for MG5) but the alloy MG5 had lower Nb/(Ti + Hf)_alloy_ ratio (0.586 for MG2 compared with 0.558 for MG5), which “brought” the alloy inside the ABC triangle (Figure 1) at the same time as it “made certain” the formation only of hexagonal γNb_5_Si_3_ (in the alloy MG2 both tetragonal and hexagonal Nb_5_Si_3_ were formed). We do not know if weight loss at 800 °C is typical of alloys near the corner C of the (Al/Si)_alloy_ versus Nb/(Ti + Hf)_alloy_ map. This is worth further investigation.

Like the alloy MG7, at 800 °C the oxidation data of the alloys MG5 and MG6 exhibited “sudden” changes in weight. However, in the latter two alloys these changes were different and occurred more often and were more severe in the alloy MG6 (Figure 7). According to the GXRD data (Figure S6 in the Supplemental data), the scale formed on each alloy contained the same oxides including the aluminium titanate TiAl_2_O_5_ plus SiO_2_ in the case of the alloy MG6 (and MG7 [3]). In the alloy MG6 the intermetallic compound TM_9_Si_7_Al_4_ was formed (Table 1). In the alloys MG5 and MG6 below the scale the TiAl_2_ compound was observed together with the TMAl_3_ aluminide. Furthermore, in the alloy MG5 the Ti_2_Al_5_ and TM_2_Si_1.5_Al compounds were also observed but not in the alloy MG6 where instead the TM_8_Al_11_Si_3_ was formed (see Section 4.2). In other words, there were differences in the substrate microstructure just below the scale formed on each alloy. The coefficient of thermal expansion (CTE) of SiO_2_ is in the range of CTE values of the oxides and phases of interest (see Table 2 in [3]) and the aluminium titanate has a very anisotropic CTE [3]. There is no data for the CTE of the other newly formed compounds and for all the compounds as their chemical compositions change (Figure 4). The “sudden” changes in weight at 800 °C observed in the alloy MG6 were attributed to the aforementioned compounds. During oxidation the formation of TiAl_2_ and Ti_2_Al_5_ was accompanied with volume changes and resulted to cracking of “composite” silicide grains, as was the case during each heat treatment (see Section 5.2.2) but these stresses may have also caused damage to the oxide scale.

The alloy MG7 also did not pest even though its microstructure was cracked at 800 °C [3] like those of the alloys MG5 and MG6. At 800 °C the aluminides and “composite” silicide grains present in the microstructures of the three alloys had different Nb/Ti, Nb/(Ti + Hf), Al/Ti and Al/Si ratios (Figure 4) owing to the different alloy compositions. The differences in chemical composition of the above compounds and of the ternary compounds observed in the alloys would be expected to have an effect on their oxidation behaviour and CTE values. The non pesting of the alloys was attributed to the presence of non-pesting intermetallic compounds in their microstructures. 

The weight change curves of the alloys MG5 and MG6 at 1200 °C were smoother compared with the alloy MG7 [3], particularly in the case of the alloy MG5 (Figure 8). All three alloys formed about 5 μm thick continuous well adhering alumina scales (Figures 10 and 13d in [3]) on top of aluminides and silicides (Figure 10). The oxidation behaviour of the alloys MG5 and MG6 at 1200 °C was attributed to the type of intermetallic alloys that were present in their microstructures and their oxidation behaviour, as discussed in [3]. The quick rise in weight gain of the alloy MG6 at the early stages (t < 10 h) of oxidation (Figure 8c) was attributed to the incipient melting. According to the GXRD data (Figure S7 in the Supplemental data) the same oxides were present in the scales formed on both alloys (and the alloy MG7 [3]) at this temperature. The TiAl_2_ was observed together with TMAl_3_ below the scale. Inside the tri-aluminide there was precipitation of a new bright contrast phase (Figure 6 and Figure 10b), which could be Si rich [47] or Ti_5_Si_4_ [49]. The only difference between the two alloys was the presence of the intermetallic compound TM_9_Si_7_Al_4_ (Table 1) and the TM_2.3_AlSi_0.3_ just below the scale in the alloy MG6. There is no CTE data for the latter compound. 

### 5.4. Comparison with High Entropy Alloys and Nb-Silicide Based Alloys

The parameters VEC, Δχ and δ (related to atomic size) of the alloys MG5 and MG6 respectively were in the ranges 3.791 < VEC < 3.935, 0.151 < Δχ < 0.166, 9.3945 < δ < 10.29 and 3.728 < VEC < 3.762, 0.148 < Δχ < 0.152, 9.05 < δ < 9.324. Compared with HEAs with bcc solid solution and intermetallics, only the VEC values of both the alloys were outside the range of reported values. Compared with Nb-silicide-based alloys only the VEC values of both the alloys were outside the reported range, and remarkably, the Δχ values of both alloys were within the range of “forbidden” values for the Nb solid solution [9,54]. The Figure 1 is a map for αAl_2_O_3_ forming multi-principle element Nb-Ti-Si-Al-Hf alloys or αAl_2_O_3_ forming complex concentrated Nb-Ti-Si-Al-Hf alloys.

### 5.5. “Layered” Structures?

**Figure 11:**
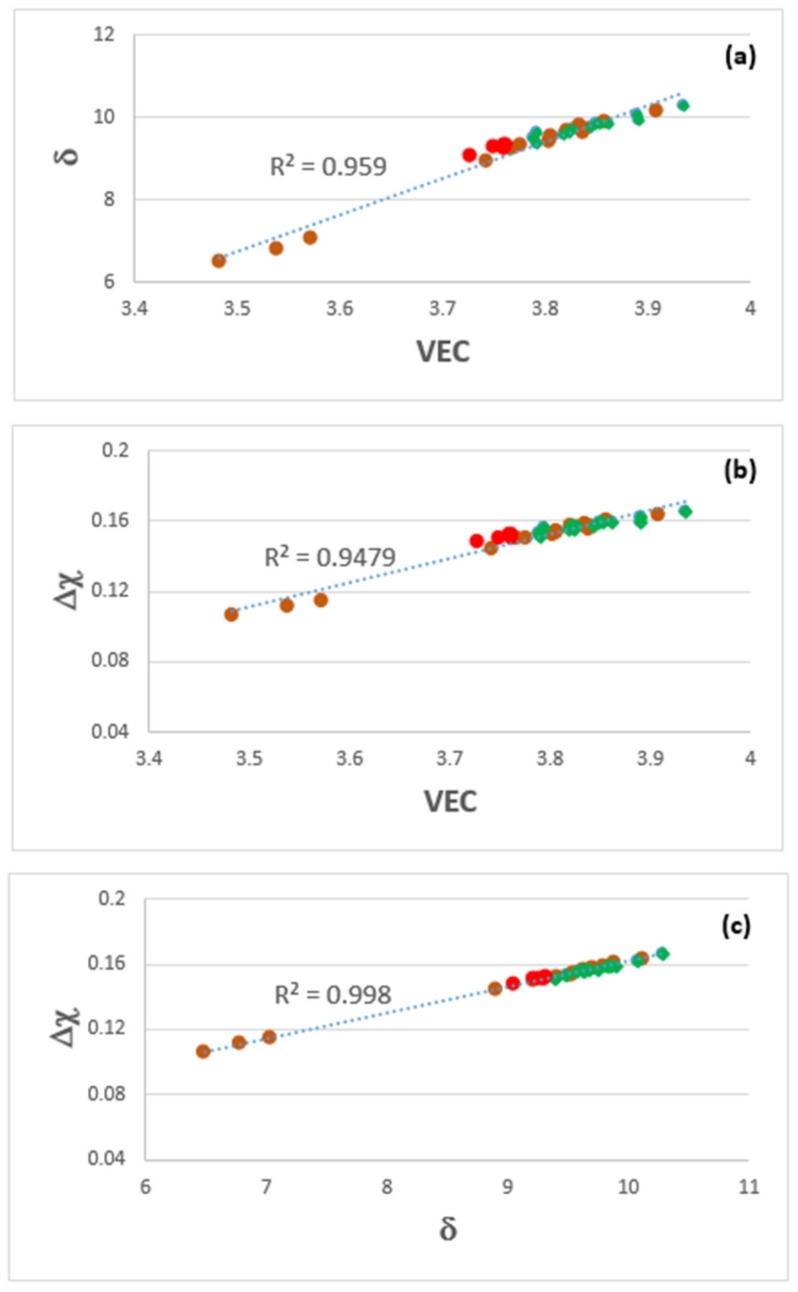
Plots of the parameters VEC, Δχ and δ for the alloys MG5 (green), MG6 (red) and MG7 (brown). (**a**) δ versus VEC, (**b**) Δχ versus VEC and (**c**) Δχ versus δ. The R^2^ values correspond to all the data. For the R^2^ values of the data only for the alloy MG7 see [3].

**Figure 12:**
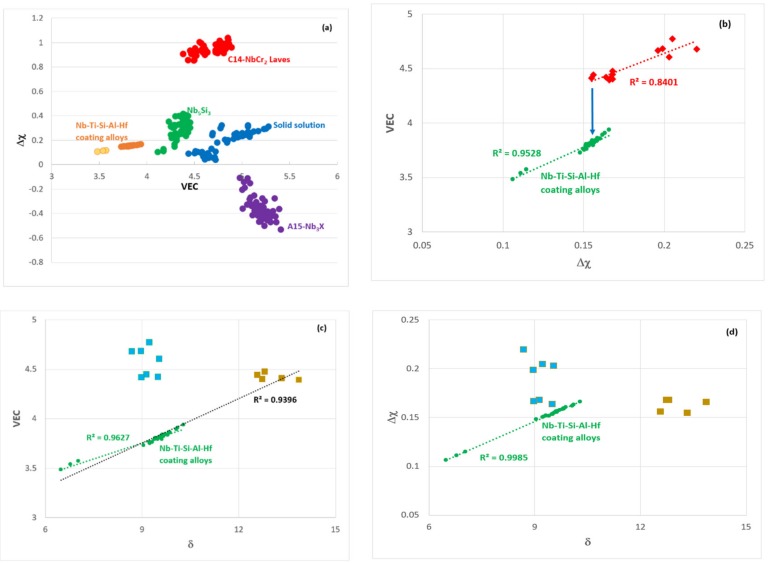
(**a**) VEC versus Δχ map of phases in Nb-silicide based alloys [27] showing the location of Nb-Ti-Si-Al-Hf coating alloys. For non-pesting alloys [54] and Nb-Ti-Si-Al-Hf coating alloys (**b**) VEC versus Δχ map, (**c**) VEC versus δ map and (**d**) Δχ versus δ map. In (c) and (d) gold colour for B containing alloys, blue colour for Sn and/or Ge containing alloys, in (b) red colour for B, Sn and Ge containing alloys. In (a) light orange colour is for zone A in layered coating alloys [3].

Plots of the parameters VEC, Δχ and δ of the alloys MG5, MG6 and MG7 are shown in the Figure 11. It should be noted that the alloy MG7 exhibited “layered” structure and “sampled” wider ranges of values of the parameters VEC, Δχ and δ [3]. It is remarkable that the data for all three alloys exhibits very good fit (R^2^ > 0.95). Figure 12a shows the location of Nb-Ti-Si-Al-Hf coating alloys in the VEC versus Δχ map of the key phases in Nb-silicide based alloys [27], i.e., in the substrates of the coatings. The coating alloys are to the left of Nb_5_Si_3_, owing to their low VEC values (see previous section). Maps of the parameters VEC, Δχ and δ for non-pesting Nb silicide based alloys and Nb-Ti-Si-Al-Hf coating alloys are shown in the Figure 12b–d. The former alloys were included in the alloys that were studied in [54]. Figure 12b shows similar VEC versus Δχ trends for non-pesting (substrate) alloys and coatings with a step change (indicated by the arrow) in the value of VEC of the latter. The data for non-pesting B containing alloys fits well with the data for coatings (R^2^ = 0.9396), see Figure 12c, but not the data in the Δχ versus δ map (Figure 12d). Is it likely that Nb-(14-24)Si-(10-26)Ti-X-Y-B (at.%) (X = simple metal, Y = refractory metal) silicide based substrate alloys might be well matched (compatible) with αAl_2_O_3_ forming Nb-Ti-Si-Al-Hf coatings? This is worth further investigation. 

The alloy MG7 formed a “layered” structure [3]. Would it be possible to form “layered” structures with compositions close to those of the alloys MG5, MG6 and MG7 (keeping in mind that the alloy MG6 suffered from incipient melting)? In the next section we shall consider further how compositions could be selected that are suitable for bond coat(s) that do not pest, might be “layered” and form αAl_2_O_3_.

#### 5.5.1. Selection of Bond Coat Alloys

**Figure 13:**
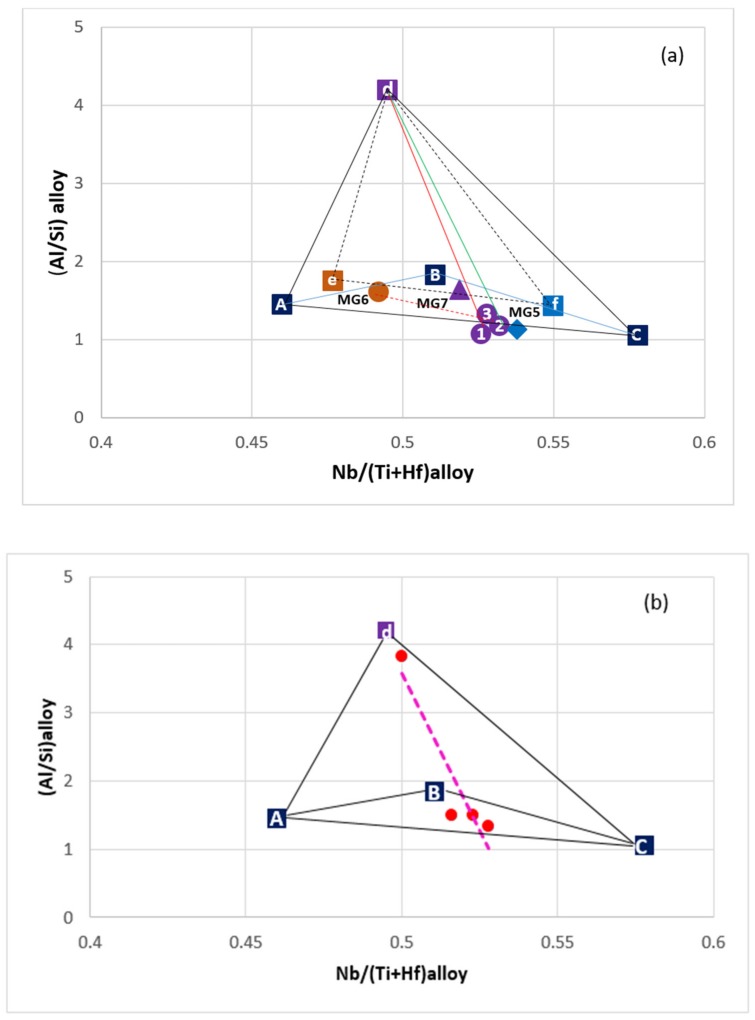
(Al/Si)_alloy_ versus [Nb/(Ti + Hf)]_alloy_ maps for Nb-Ti-Si-Al-Hf alloys (see text). (**a**) The alloys Nb_1.45_Si_2.7_Ti_2.25_Al_3.25_Hf_0.35_ (MG5), Nb_1.35_Si_2.3_Ti_2.3_Al_3.7_Hf_0.35_ (MG6) and Nb_1.3_Si_2.4_Ti_2.4_Al_3.5_Hf_0.4_ (MG7) are shown in the map for their actual compositions. Brown MG6, purple MG7 and blue MG5. The triangle def is defined by the highest (Al/Si)_alloy_ values and corresponding Nb/(Ti + Hf)_alloy_ ratios due to macrosegregation in the alloys MG5, MG6 and MG7 (the strongest segregating elements in these alloys were Al and Si). The numbers 1 to 3 are for the top (1), bulk (2) and zone B (3), and d is for the zone A of the alloy Nb_1.3_Si_2.4_Ti_2.4_Al_3.5_Hf_0.4_ (MG7) [3]. For the triangle ACd see text. (**b**) shows the bond coat alloys (red circles) in the ACd triangle.

Figure 13 shows the (Al/Si)_alloy_ versus [Nb/(Ti + Hf)]_alloy_ map for Nb-Ti-Si-Al-Hf alloys. The triangle ABC is the same as in Figure 1. If the latter were to be compared with the Figure 13a, the significant “shift” in the position of the alloy MG7 should be noticed because of its “layered” structure. It should also be noted that the values of the [Nb/(Ti + Hf)]_alloy_ and (Al/Si)_alloy_ ratios for the actual composition of the alloy MG2 were 0.67 and 1.3 and fall outside (not shown) the triangles ABC and ACd in the Figure 13a. In the latter the triangle ACd is “defined” by NICE (side AC) and the structure of the alloy MG7 (i.e., the layered structure, Zone A, point d). The triangle ACd is for alloys of the Nb-Ti-Si-Hf-Al system that have no solid solution, gain very low weight per unit area (<3 mg/cm^2^) in isothermal oxidation at 800 and 1200 °C and form αAl_2_O_3_ in their scales. In Figure 13a (i) the “layered” structure that was formed in the alloy MG7 [3] is represented by the continuous red line and (ii) the red dotted line shows a “pathway” in the map for (possibly) a “layered” structure with compositions corresponding to those of MG5, (the numbers) 2 and 3 (see figure caption) and MG6 in the ABC triangle. In such a “layered” structure it is likely that compositions near MG6 might suffer incipient melting (exhibit liquation) at 1200 °C. Another possible “layered” structure could be formed along the pathway indicated by the green line in the triangle ACd with compositions corresponding to those of MG5, (the numbers) 2 and 3 and (the letter) d (see figure caption). In other words, it is suggested that actual compositions that fall near M6, i.e., near the AB side of the triangle ABC in Figure 13 should be avoided because they might be prone to liquation. Further research is required to verify this.

**Figure 14:**
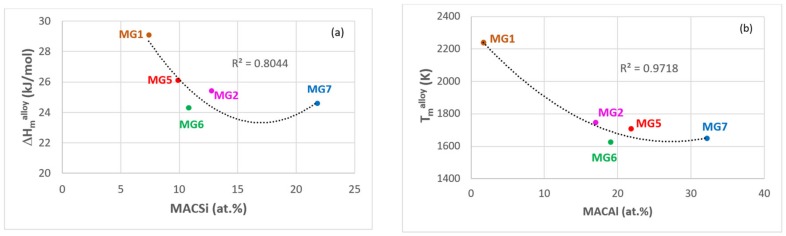
Relationships between the parameter (**a**) ΔH_m_^alloy^ versus MACSi and (**b**) T_m_^alloy^ versus MACAl.

Can alloy design and selection benefit from data about the chemical inhomogeneity (macro-segregation) in alloys of the Nb-Ti-Si-Al-Hf system? Remarkably, when data for MACSi, MACAl and MACTi was plotted against the parameters that can account for the macrosegregation in Nb-Si based alloys [34], (a) the fit of data was good and (b) the data exhibited maxima or minima (for example, see Figure 14). The values of the parameters corresponding to the maxima or minima in the data were used to calculate the average composition of an alloy by solving a 5x5 system of linear equations. An example of the calculation of the composition of an alloy aNb + bTi + cSi + dAl + eHf (at.%) is given in the Appendix A.

**Figure 15:**
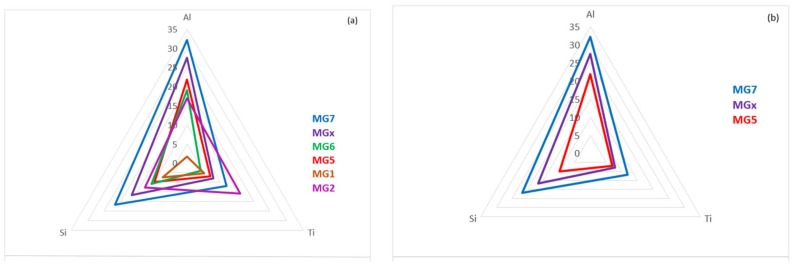
Macrosegregation map of Al, Si and Ti for the Nb-Ti-Si-Al-Hf system constructed using data for the alloys MG1, MG2, MG5, MG6, MG7 and MGx (see text). (**a**) All alloys, (**b**) alloys MG5, MGx, MG7.

Figure 15a is a map defined by the macrosegregations of Al, Si and Ti in the alloys of the Nb-Ti-Si-Al-Hf system that have been reported by our group [3,35] and shows macrosegregation “isolines”. Figure 15b excludes the alloys MG1 [35] and MG2 [3] owing to their poor oxidation and the alloy MG6 because of incipient melting at 1200 °C. Figure 15 should not be compared with the Figure 1, Figure 2 and Figure 13. In the Figure 15 the alloy MGx has the composition 10.1Nb-22.6Ti-24.3Si-36.6Al-6.4Hf (at.%), which is the average of the compositions that were calculated as discussed above (see Appendix A), and its corresponding values of MACAl, MACSi and MACTi (the corners of the purple triangle) are the values that were calculated from the minima or maxima of data like that shown in Figure 14. 

It is suggested that bond coat (BC) structure(s) suitable for a coating system [3] could be formed with compositions along the continuous green line in Figure 13a and chemical in-homogeneities of Al, Si and Ti that fall in-between the blue and red triangles in the map shown in Figure 15b, where the purple triangle corresponds to the alloy MGx (see above). An example of a multi-material bond coat selected using NICE [27] and knowledge about the processing of Nb-silicide based alloys [55,56] is given below. The bond coat could include one, some or all or average(s) of two or more of the following compositions. However, chosen average compositions should fall in the map shown in Figure 13.

Bond Coat: 11.1Nb-18.5Ti-13.8Si-52.9Al-3.7Hf//13.5Nb-22.7Ti-24.4Si-36.5Al-3.9Hf//13.3Nb-22.4Ti-24.3Si-36.6Al-3.4Hf//11.8Nb-25.9Ti-25.3Si-33.6Al-3.4Hf.

The (Al/Si)_alloy_ and Nb/(Ti + Hf)_alloy_ ratios of the BC fall in the ACd triangle (see Figure 13b). Furthermore, the differences between the maximum and minimum values of the elements Al, Si and Ti (i.e., C_max_^i^ – C_min_^i^ (I = Al,Si,Ti)), which is the definition of macrosegregation of element i fall in the space between the blue and red triangles in Figure 15.

It is also suggested that the key microstructure for oxidation resistance consists (i) of “composite” silicide grains that “protect” the susceptible to pesting Nb_5_Si_3_ with higher order (i.e., richer in Si) Ti silicides that (a) do not pest, (b) exhibit solubility for Al, in particular the TiSi and (c) have superior oxidation than Nb_5_Si_3_ when contaminated by oxygen [51], and (ii) of tri-aluminides with very low TM/Ti ratio forming in-between the “composite” silicide grains. 

The Nb/(Ti + Hf)_alloy_ ratio is key for the formation of hexagonal γNb_5_Si_3_ and the value of the Nb/(Ti + Hf) ratio is also important for the stability of γNb_5_Si_3_ (see Section 2). For the formation of “composite” silicide in the microstructure (a) the value of the Nb/(Ti + Hf) ratio of the γNb_5_Si_3_, Ti_5_Si_4_ and TiSi silicides must be less than one and (b) the alloy composition must lie inside the triangle ABC in Figure 1 and inside the map shown in Figure 2.

### 5.6. Suggestions for Future Work

Isothermal oxidation is a measure of the performance of an alloy but it is at best a poor yardstick. Evaluation of oxidation resistance must also consider the ability of the scale to resist the thermally induced stresses associated with cyclic behaviour. First one would need to find out whether the alloy MG5 has good oxidation under cyclic conditions. Then, one would need (i) to select (a) a substrate alloy with inherent oxidation resistance and balance of mechanical properties, (b) a coating process and processing conditions to deposit a (single or multi-alloy) bond coat, (ii) to evaluate the oxidation of the substrate/BC system under (c) cyclic conditions, (iii) to get insight into the mechanism(s) of the interaction between the coating and the substrate and thus to assess (iv) the stability of the substrate/BC interface and (v) the contamination of the substrate by interstitials. It is hoped that the work presented in this paper will inspire new research on the development of coatings for Nb-silicide based alloys.

## 6. Summary

A (Al/Si)_alloy_ versus Nb/(Ti + Hf)_alloy_ map, which can be considered to be a map for multi-principle element or complex concentrated Nb-Ti-Si-Al-Hf alloys, and a [Nb/(Ti + Hf)]_Nb5Si3_ versus [Nb/(Ti + Hf)]_alloy_ map were used to select the alloys Nb_1.45_Si_2.7_Ti_2.25_Al_3.25_Hf_0.35_ (MG5) and Nb_1.35_Si_2.3_Ti_2.3_Al_3.7_Hf_0.35_ (MG6) that were expected (i) not to pest, (ii) to form αAl_2_O_3_ scale at 1200 °C, (iii) to have no solid solution, (iv) to form only hexagonal Nb_5_Si_3_ and (v) to have microstructures consisting of hexagonal Nb_5_Si_3_, Ti_5_Si_3_, Ti_5_Si_4_, TiSi silicides, and tri-aluminides and Al rich TiAl. Both alloys met (i) to (v). The alumina scale was able to self-heal at 1200 °C. Liquation in the alloy MG6 at 1200 °C was linked with the formation of a eutectic like structure and the TiAl aluminide in the cast alloy.

Key to the oxidation of the alloys was the formation (i) of “composite” silicide grains in which the γNb_5_Si_3_ core was surrounded by the higher order (i.e., richer in Si) Ti_5_Si_4_ and TiSi silicides (a) that do not pest, (b) exhibit solubility for Al, in particular the TiSi and (c) have superior oxidation than Nb_5_Si_3_ when contaminated by oxygen, (ii) of tri-aluminides with very low Nb/Ti ratio forming in-between the “composite” silicide grains and high Al/Si ratio, particularly at 1200 °C.

## Figures and Tables

**Table 1:** Phases in the microstructures of the cast and heat treated alloys MG5, MG6 and MG7 [3] confirmed by XRD and quantitative EDS.

Phase	As Cast	Heat Treated		As Cast	Heat Treated		As Cast	Heat Treated	
		800 °C	1200 °C		800 °C	1200 °C		800 °C	1200 °C
	Alloy								
	MG5			MG6			MG7		
TMAl_3_	X	X	X	X	X	X	X	X	X
Ti_5_Si_4_	X	X	X	X	X	X	X	X	X
TiAl		X	X	X	X	X		X	X
γNb_5_Si_3_	X	X	X	X	X	X	X	X	X
TiSi	X	X	X	X	X	X	X	X	X
TM_9_Si_7_Al_4_					X	X			

## Supplementary Materials

The following are available online at https://www.mdpi.com/1996-1944/12/5/759/s1, Figure S1: Average concentrations of elements in different areas of the as cast and heat treated alloys (a) MG5 and (b) MG6; Figure S2: XRD data of the cast alloys (a) MG5 and (b) MG6; Figure S3: Average concentrations of elements in different intermetallic compounds in the as cast and heat treated alloys MG5 and MG6. (a) TMAl3, (b) γNb5Si3, (c) Ti5Si4, (d) TiSi, (e) TiAl; Figure S4: XRD data of the heat treated alloys at 800 °C (a) MG5 and (b) MG6; Figure S5: XRD data of the heat treated alloys at 1200 °C (a) MG5 and (b) MG6; Figure S6: Glancing angle XRD data at 800 °C (a) MG5, (b) MG6; Figure S7: Glancing angle XRD data at 1200 °C (a) MG5, (b) MG6.

## Appendix A

Calculation of the composition of an alloy aNb + bTi + cSi + dAl + eHf (at.%)

a + b + c + d + e = 1 (A1)

aT_m_^Nb^ + bT_m_^Ti^ + cT_m_^Si^ + dT_m_^Al^ + eT_m_^Hf^ = T_m_^alloy^ = 1629.3 K (A2)

aH_m_^Nb^ + bH_m_^Ti^ + cH_m_^Si^ + dH_m_^Al^ + eH_m_^Hf^ = ΔH_m_^alloy^ = 24.7 kJ/mol (A3)

aT_m_^Nb^ + bT_m_^Ti^ + eT_m_^Hf^ = T_m_^sd^ = 877.86 K (A4)

cH_m_^Si^ + dH_m_^Al^ = ΔH_m_^sp^ = 16.13 kJ/mol (A5)

where T_m_^i^ is the melting temperature (K) of element i, H_m_^i^ is the enthalpy of melting (kJ/mol) or element i, T_m_^sd^ is the “melting temperature” of elements of sd electronic configuration and ΔH_m_^i^ is the “enthalpy of melting” of elements of sp electronic configuration (see [34]), where i = Al,Hf,Nb,Si,Ti. Substitution of values of T_m_^i^ and H_m_^i^ gives a = 10.47, b = 22.73, c = 24.35, d = 36.52 and e = 5.93 (at.%). Note that T_m_^alloy^ = 1629.3 K corresponds to the minimum in Figure 13b. Another 5 x 5 system of linear equations consisting of Equations 1 to 3 and those for T_m_^sp^ = 751.44 K and ΔH_m_^sd^ = 8.58 kJ/mol gives a = 9.93, b = 22.39, c = 24.32, d = 36.57 and e = 6.79 (at.%), not significantly different from the previously calculated concentrations.
