# Supplementary material for: Microstructures and Isothermal Oxidation of the Alumina Scale Forming Nb1.45Si2.7Ti2.25Al3.25Hf0.35 and Nb1.35Si2.3Ti2.3Al3.7Hf0.35 Alloys
Source: Materials (Basel). 2019 Mar 5;12(5):759. doi: 10.3390/ma12050759 (PMC6427440; doi:10.3390/ma12050759)
Supplement: Supplementary file 1 [file materials-12-00759-s001.pdf]

materials

Supplementary

Microstructures and Isothermal Oxidation of the Alumina Scale Forming $Nb_{1.45}Si_{2.7}Ti_{2.25}Al_{3.25}Hf_{0.35}$ and $Nb_{1.35}Si_{2.3}Ti_{2.3}Al_{3.7}Hf_{0.35}$ Alloys

Mohammad Ghadyani, Claire Utton and Panos Tsakiropoulos *

Department of Materials Science and Engineering, Sir Robert Hadfield Building, University of Sheffield, Mappin Street, Sheffield S1 3JD, UK; m.ghadyani@sheffield.ac.uk (M.G.); c.utton@sheffield.ac.uk (G.U.)

* Correspondence: p.tsakiropoulos@sheffield.ac.uk

Received: 21 January 2019; Accepted: 1 March 2019; Published: 7 March 2019

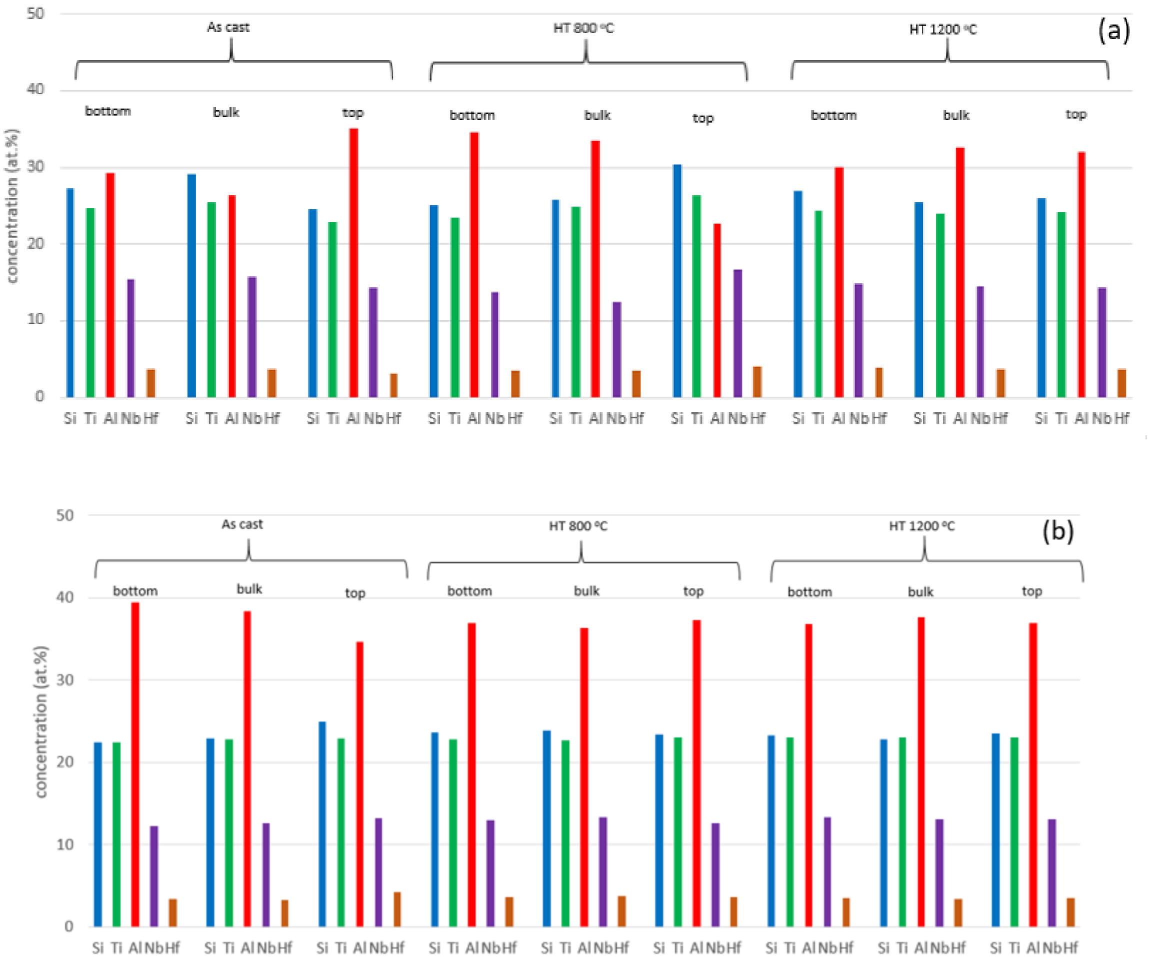

Figure 1. Average concentrations of elements in different areas of the as cast and heat treated alloys (**a**) MG5 and (**b**) MG6.

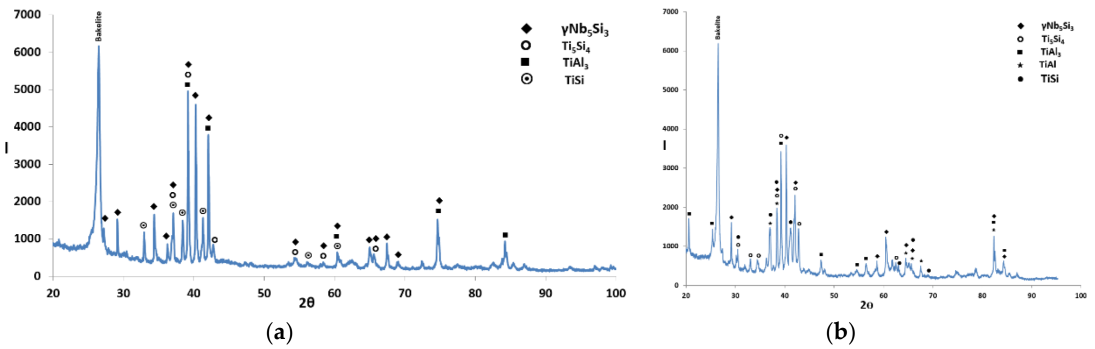

Figure 2. XRD data of the cast alloys (**a**) MG5 and (**b**) MG6.

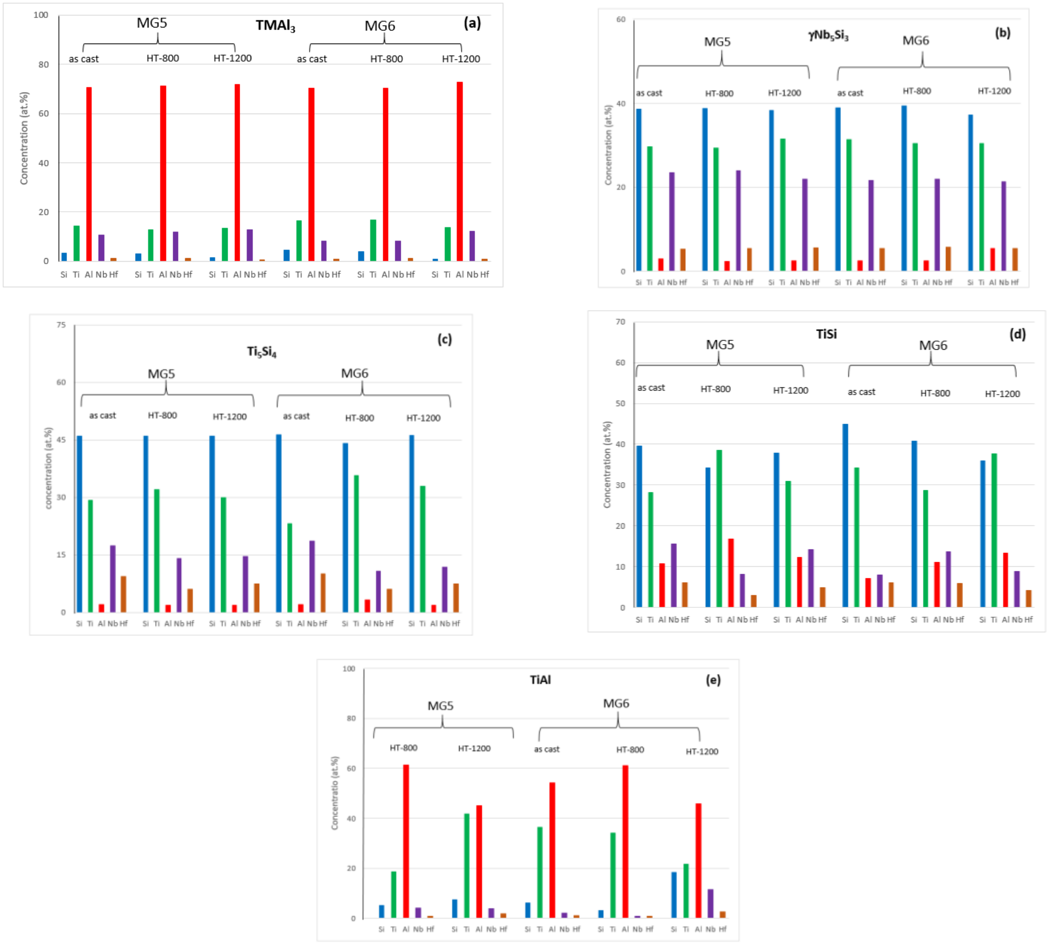

Figure 3. Average concentrations of elements in different intermetallic compounds in the as cast and heat treated alloys MG5 and MG6. (**a**) TMAl3, (**b**) γNb5Si3, (**c**) Ti5Si4, (**d**) TiSi, (**e**) TiAl.

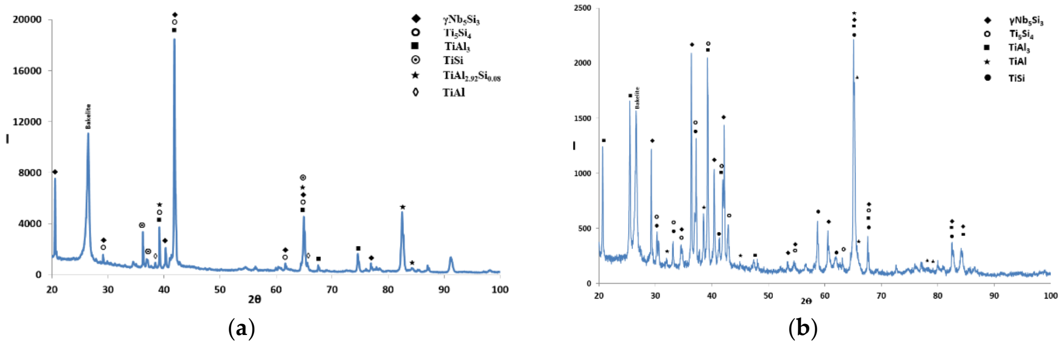

Figure 4. XRD data of the heat treated alloys at 800 °C (**a**) MG5 and (**b**) MG6.

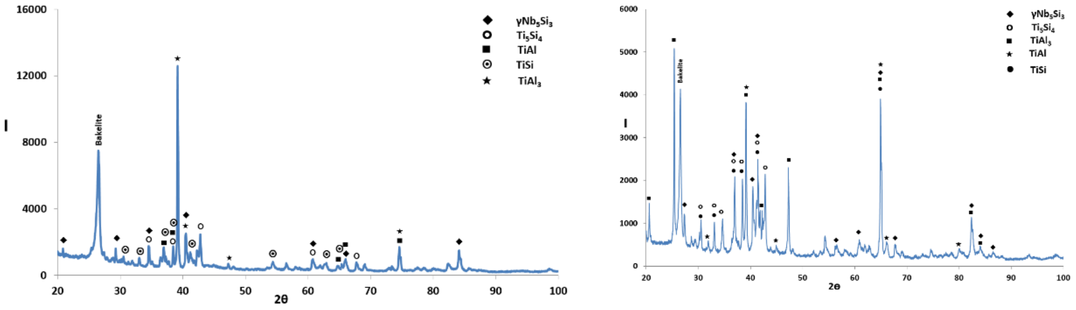

(**a**) (**b**)

Figure 5. XRD data of the heat treated alloys at 1200 °C (**a**) MG5 and (**b**) MG6.

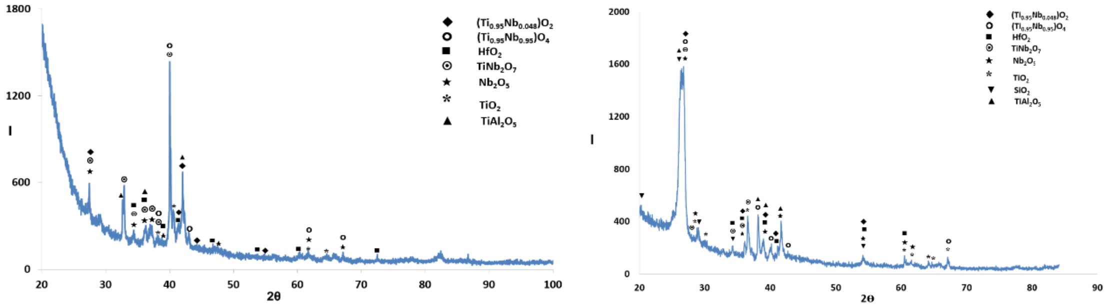

(**a**) (**b**)

Figure 6. Glancing angle XRD data at 800 °C (**a**) MG5, (**b**) MG6.

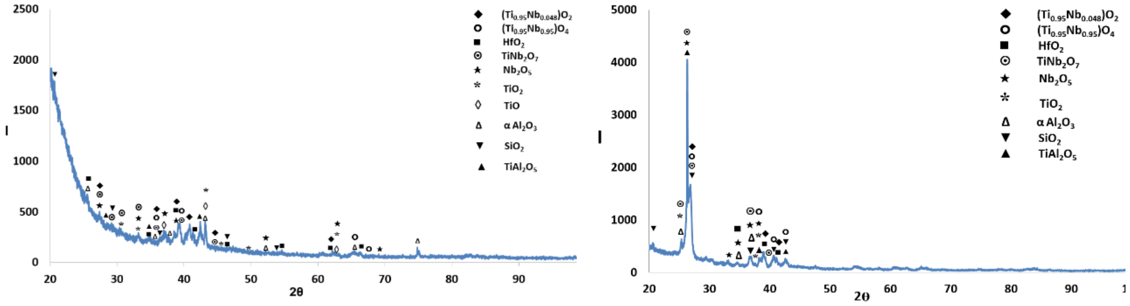

(**a**) (**b**)

Figure 7. Glancing angle XRD data at 1200 °C (**a**) MG5, (**b**) MG6.

© 2019 by the authors. Licensee MDPI, Basel, Switzerland. This article is an open access article distributed under the terms and conditions of the Creative Commons Attribution (CC BY) license (http://creativecommons.org/licenses/by/4.0/).
